# Length-based assessment of five small pelagic fishes in the Senegalese artisanal fisheries

**DOI:** 10.1371/journal.pone.0279768

**Published:** 2022-12-30

**Authors:** Bocar Sabaly Baldé, Patrice Brehmer, Penda Diop Diaw

**Affiliations:** 1 Institut Sénégalais de Recherche Agricole, ISRA, Centre de Recherche Océanographique de Dakar-Thiaroye, CRODT, Dakar, Sénégal; 2 IRD, Univ Brest, CNRS, Ifremer, UMR Lemar, Dakar, Sénégal; 3 Commission Sous Régionale des Pêches, CSRP, Secrétariat Permanent de la CSRP, Dakar, Sénégal; MARE – Marine and Environmental Sciences Centre, PORTUGAL

## Abstract

Fisheries management is an important strategy for ensuring sustainable use of resources. However, in West Africa, in the absence of quality data for many stocks and effective stock assessment models, the cases where this has been truly successful are notable for their rarity. In West Africa, small pelagic fish are of great socio-economic importance, as well as good indicators of fish stressors. Here, historical data (2004–2019) of five small pelagic species (*Sardina pilchardus*, *Ethmalosa fimbriata*, *Trachurus trecae*, *Scomber colias* and *Mugil cephalus*) were collected in Senegalese waters. The *B/B*_*MSY*_ results showed stocks to be collapsed (*B/B*_*MSY*_ = 0.13 and 0.1 for *M*. *cephalus* and *S*. *pilchardus*, respectively) and heavily overfished (*B/B*_*MSY*_ = 0.24; *E*. *fimbriata*). Only *S*. *colias* and *T*. *trecae* stock were considered to be in good condition (*B/B*_*MSY*_ = 1.7 and 1.4 respectively). The *L*_*c*_*/L*_*c_opt*_ ratio was ≤ 1 for *E*. *fimbriata* and *M*. *cephalus*, suggesting that the individuals caught for these species were too small. To reverse these bad stock statuses, catching individuals at *L*_*c_opt*_, 25, 21, 43 and 18 cm for *S*. *colias*, *E*. *fimbriata*, *M*. *cephalus* and *S*. *pilchardus*, respectively should be a natural guarantee against recruitment failure and allow individuals to ensure the long-term survival of populations, in a context of data poor fisheries. In conclusion, this study shows that, despite limitations, the LBB model can provides indicators of stock status for species to encourage management measures, especially in data poor countries. It is hoped that these results can help to better assess many stocks currently considered too data poor to be assessed or at least encourage data collection effort on stocks discerned as in bad or critical status.

## 1. Introduction

In West Africa, the fishing sector plays an important role by providing food security and nutrition [1, 2]. It’s also the main resource for traditional processing activities (drying or smoking) and plays a vital role in the diet of population [3]. However, with poorly adapted and ineffective fisheries management policies, this sector is confronted with the effects of overfishing and the collapse of fisheries in terms of local consumption, food security, and economic value [2, 4, 5]. Attempts to regulate fisheries in the region through the establishment of marine protected areas and gear restrictions have been limited by the lack of scientific data and inadequate infrastructure and human capacity to effectively monitor and assess marine resources [5, 6]. Indeed, the data available have some major drawbacks. Nominal catches are sometimes inaccurate [2, 5]. Indeed, they often contain inaccurate transcriptions of weights and sizes of individuals. Insufficient data on fishing effort, in addition to unreliable and outdated statistical data also form part of the problem [2, 5]. This affects their use in the formulation of relevant policies for the sector [2]. Consequently, management guidelines and controls must be simple, but also robust against uncertainties, as well as being proportionate to the information available [7]. Thus, it is possible to apply generic management procedures, which are not necessarily the best for a given fishery, but which might be better than taking no action [8].

Recently, several stock assessment methods have been developed and applied to many stocks with poor data [9–11]. The Length-based Integrated Mixed Effects (LIME) model uses a dynamic age-structured model and assumes that biological input parameters are known without error, length at age is normally distributed, natural mortality rates are constant over time and growth rates are constant between cohorts with the ability to account for fishing mortality [12]. However, the estimation of some parameters such as recruitment (*r*) and fishing mortality (*F/M*) may be uncertain as they are assessed over a single year of length [12]. The Catch Maximum Sustainable Yield model [13] estimates reference points (*F*_*MSY*_, *B*_*MSY*_) as well as relative stock size (*B/B*_*MSY*_) and exploitation (*F/F*_*MSY*_) while the Depletion-Based Stock Reduction Analysis model [14] estimates maximum sustainable yield. However, the prediction of the CMSY method is only accurate when validated with real data from simulated stocks or evaluated against the *B*_*MSY*_ estimate for real stocks, whereas the DB-SRA model is limited by the inability to cope with the uniform decrease in abundance as well as an underestimation of the overfishing limit values depending on stock characteristics [14]. The LBB model is a Length-Based Bayesian (LBB) biomass estimate based on processes for analysing length frequency (LF) or width frequency data of fish or invertebrate populations [11]. According to Froese et al. [11], this model works for species that grow throughout their lifetime. It estimates asymptomatic length (*L*_*∞*_), length at first capture (*L*_*c*_), natural mortality (*M/K*) and fishing mortality (*F/K*). The LBB model is increasingly applied in Asian fisheries [15, 16]. But also, it is considered as a promising method in international commissions such as ICCAT [17, 18]. Wang et al. [15], who used the LBB model on the species *Portunus trituberculatus* in China, compared the results with other research and showed that this methodology can be used in the case of data-poor stocks. Indeed, this model also provides a comparison of the current length at first capture (*L*_*c*_) versus the length (*L*_*c_opt*_) that would maximize catch and biomass for a given fishing pressure [19]. It also provides estimates of relative fishing mortality (*F/M*), which can be considered a proxy for estimates of *F/F*_*msy*_ as typically presented in full stock assessments [11].

In Senegal, artisanal fishing is the sector that targets coastal pelagic resources the most because of their presence along the Senegalese coast and the fact that the resource is much more accessible to their fishing gear [2, 20, 21]. The Senegalese fishing fleet is the most important in the West African sub-region (including Nigeria and Ghana) [2]. Indeed, artisanal fishing is practiced by several fishing communities using more than twenty fishing techniques following strategies that vary seasonally according to biological and socio-economic factors [1, 5]. Fishing techniques have evolved, and the Senegalese canoe, considered as a traditional boat, has undergone a real evolution on a historical scale under the effect of an endogenous technological dynamic that responds to the multiple expected uses [20]. Fishing statistics are difficult to control because of the large number of landing points in artisanal fishing and the difficulty of obtaining detailed data on catches and fishing effort [2]. Consequently, catch per unit effort (CPUE) in artisanal fisheries is subject to multiple sources of variability due to the composite nature of the fishery [22]. In the period 2014 to 2019, an increase of 0.7% in total landings was observed between 2014 and 2015 with 574,137 tons (t) and 578,296 t, respectively. The total landings of the artisanal fishery then decreased until 2017 (525,744 and 460,445 t in 2016 and 2017, respectively) [23]. In 2018, total landings decreased by -13.7% (397,388 t) compared to 2017 [23]. However, in 2019, total landings of the artisanal marine fishery increased by 12% compared to 2018, with a production of 445,406 t [23].

**Fig 1:**
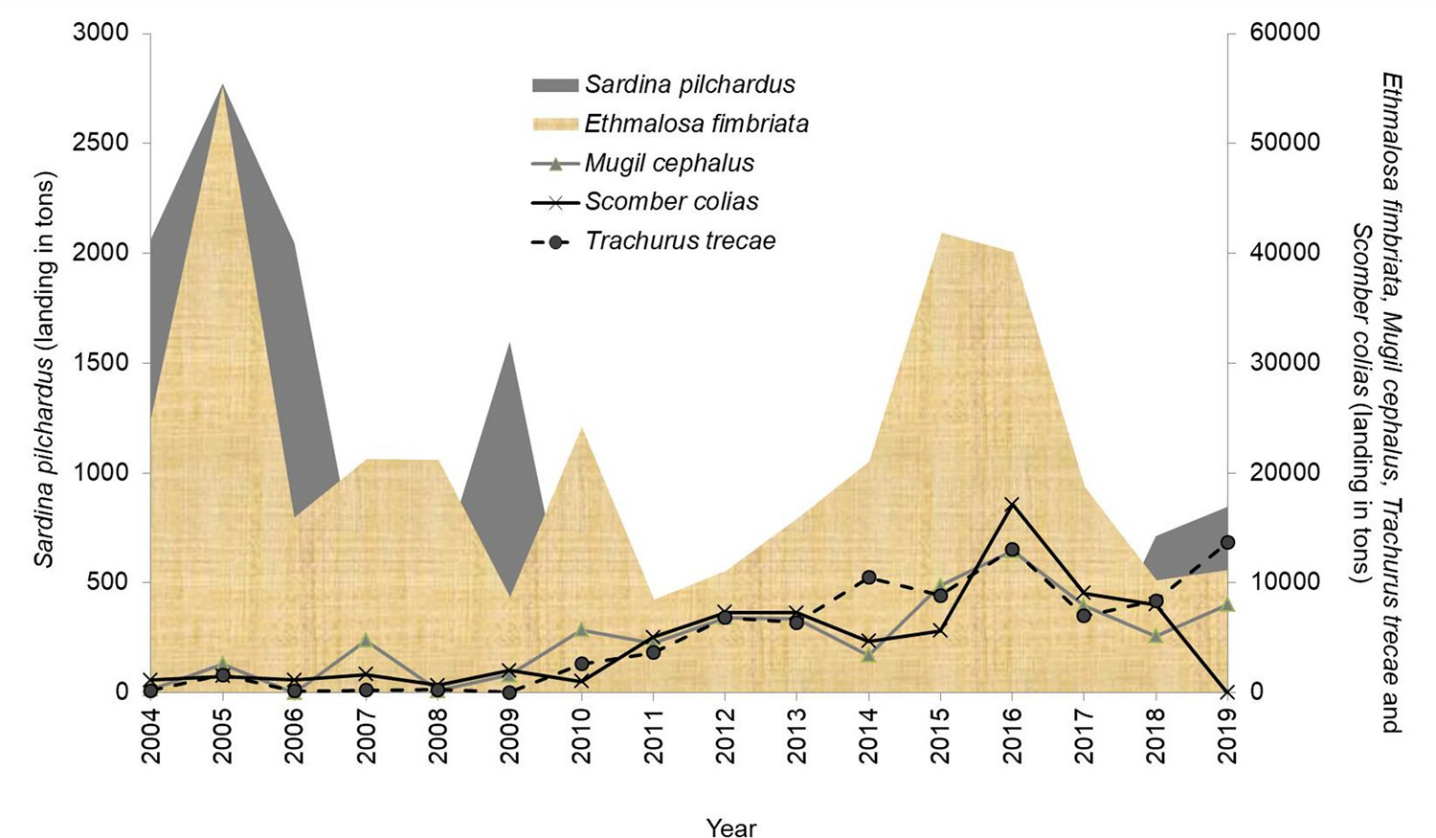
Landing of *Sardina pilchardus* (grey fill), *Ethmalosa fimbriata* (beige fill), *Mugil cephalus* (grey line with triangle), *Scomber colias* (black line with cross) and *Trachurus trecae* (black dashed line) of the artisanal fishery of Senegal (data: 2004 to 2019). Data obtained from the Centre de Recherches Océanographiques de Dakar-Thiaroye (CRODT; Senegal).

The species *S*. *pilchardus* inhabits subtropical regions (8°N– 14°N, 32°W– 43°E), occurs in fish schools [24] at high concentrations along the northwest coast of Africa and Mediterranean Sea [25]. *Sardina pilchardus* are almost exclusively caught by artisan fishers using motorized canoes with purse seines (400 to 1000 m in length) as fishing gear in Senegal (see [21, 26]). In 2005, 2006, 2009 and 2019, catches of European pilchard in Senegal (2004–2019 period) have increased considerably with estimated tonnages of 2700, 2006, 1600 and 847 t, respectively (Fig 1). *Ethmalosa fimbriata* (Bowdich, 1825), is a tropical species dependent on the estuary, distributed from Mauritania to Angola [27] and is the most common clupeidae in the brackish waters (between 5 and 90 ppt) of West African coastal estuaries [27]. The catches of *E*. *fimbriata* in Senegal (Fig 1) have fluctuated slightly over the study period (2004–2019) with peaks in 2005 (55,000 t), 2010 (24,000 t) and 2015 (42,000 tons). The range of *T*. *trecae* extends from Morocco to Angola [28]. *Trachurus trecae* is a bentopelagic species, generally found near the bottom between 20 and 100 m depth [29]. In Senegal, horse mackerel are caught by both an industrial fishery that almost stopped in April 2012 and an artisanal fishery that catches them as by-catch. Annual catches (Fig 1) show a clear increase between 2004 and 2019, marked by peaks in 2014 (10,000 t), 2016 (13,000 t) and 2019 (13,700 t). *Mugil cephalus* is distributed in the warm and temperate waters of the Atlantic, Pacific and Indian Oceans [30]. It lives abundantly in coastal marine waters, estuaries and lagoons [30]. Its range extends over the continental shelf in depths of 0–120 m. Catches of *M*. *cephalus* in Senegal have been estimated at 8,000 t in 2019 (Fig 1). *Scomber colias* is also a cosmopolitan pelagic species of medium size [31] with a highly migratory character on the continental shelf. It leads mainly a coastal pelagic life and to a lesser extent an epipelagic or mesopelagic life on the continental slope [31]. The species is mainly distributed at depths of up to 250–300 m [31]. In Senegal, *S*. *colias* is considered a by-catch by the Senegalese artisanal fleet [32]. During the period 2004–2019 (Fig 1), catches increased considerably in 2011 (5,000 t), 2015 (6,000 t), 2016 (17,000 tons) and 2017 (9,000 t). In this study, we demonstrate the applicability of the LBB model for stock assessment based on length frequencies (LF) on artisanal fisheries considered poor in data. The use of this LBB model allows the estimation of length at first capture *L*_*c*_ where 50% of the individuals are retained by the gear, natural mortality rate (*M*) relative to somatic growth rate (*M/K*) and fishing mortality rate (*F*) to somatic growth rate (*F/K*) as well as current biomass relative to unfished biomass (*B/B*_*0*_). The data required for the analysis proposed in this study are LF representative of the artisanal fishery and collected for five small pelagic species (*Sardina pilchardus*, *Ethmalosa fimbriata*, *Trachurus trecae*, *Scomber colias* and *Mugil cephalus*) in Senegalese waters from 2004 to 2019. We hope that these results can help to assess many stocks currently considered too data poor to be assessed, particularly in developing countries.

## 2. Materials and methods

### 2.1. Study area

**Fig 2:**
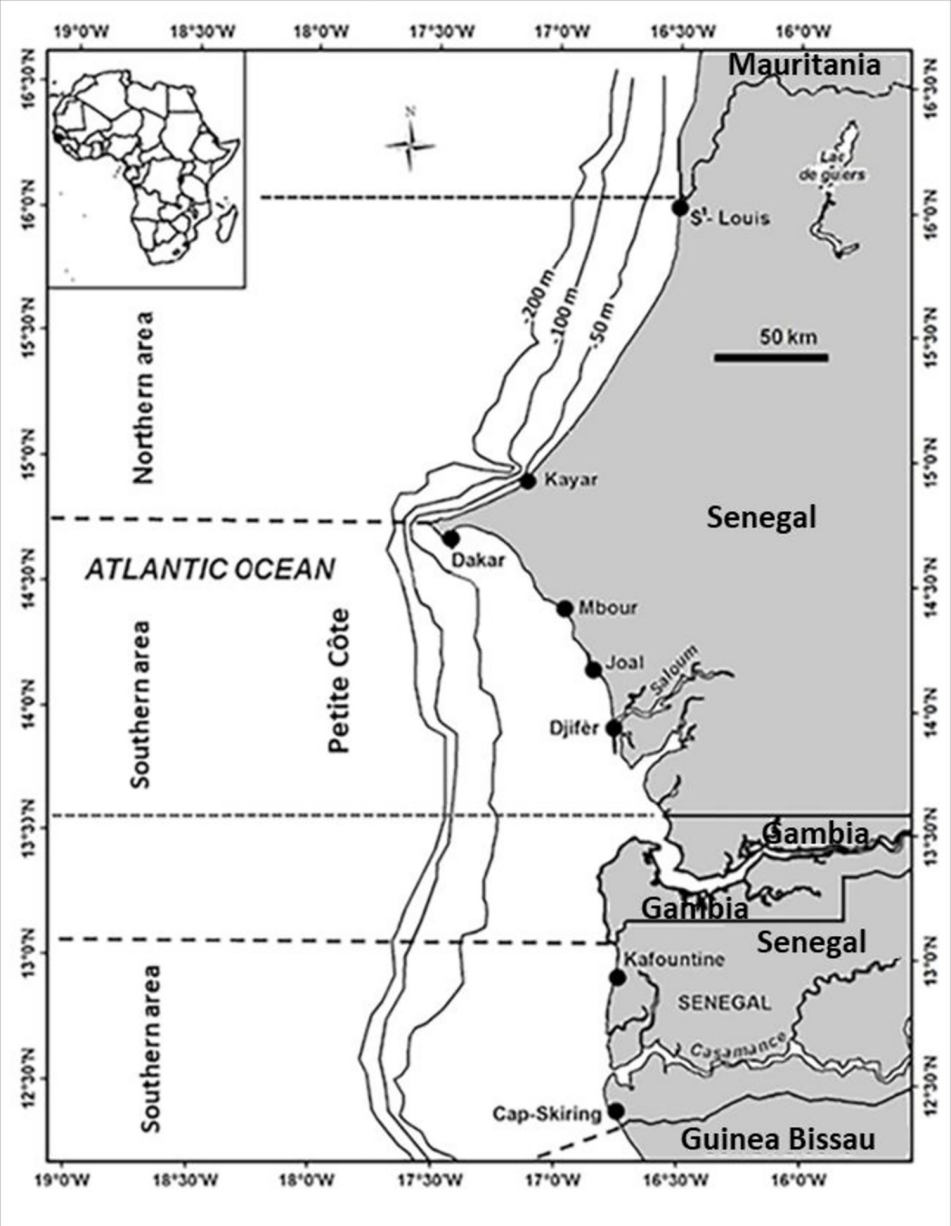
Map of the study area with the localization of sampling stations corresponding to the main landing ports along the Senegal coast. The northern section includes Saint-Louis, Kayar, and Yoff; the southern section includes the “Petite côte” (Hann, Mbour, and Joal) and Casamance (Kafountine).

The Senegalese territory covers an area of 196,722 km^2^ between 12° and 17° N, and between 11° and 18° W (Fig 2). Its continental shelf, between the shore and the 200 m isobath, covers an area of 600 km^2^, unequally distributed according to depth: 15% between 0–10 m, 49% between 10–50 m and 36% between 50–200 m [33]. At the level of Cape Vert peninsula (Dakar, Senegal), the plateau narrows considerably and the 200 m isobath is only 5 nautical miles from the coast in the south of the country, while a few dozen kilometers further north, the Kayar trench cuts deeply into it. These two obstacles, although passable by pelagic species, are responsible for certain hydroclimatic and ecological particularities in the region. Indeed, they play an important role in limiting the seasonal migration of some species (e.g. *E*. *fimbriata*; see [1]) towards the south [34].

### 2.2. Biological data

Length composition data of five species of small pelagic fish (Table 1, Supplementary materials A in S1 File) were obtained from fish in the seven main artisanal fishing ports located along the Senegalese coast (Table 1, Fig 2). Three landing sites (Kayar, Saint-Louis and Yoff) are located in the Northern part. The other landing sites (Hann, Mbour, Joal and Kafountine) are located along the Southern part. Length data were collected at random approximately 5 days per week. Total length (TL in cm) of fish was measured to the nearest cm, to calculate size-frequency distributions and to estimate growth parameters.

**Table 1:** Basic information for the five studied fish stocks.

Family	Common name (scientific name)	Location	Landing port	TL range	N	Length Type	*L*_*50*_	*L*_*∞*_ prior	*M/K* prior	Time period
				(cm)			(cm)	(cm)		
Scombrinae	*Scomber colias*	Northern Area (from 16° 04’ N to 14° 36’ N) and Southern Area (from 14° 36’ N to 13° 36’ N)	Kayar, Saint-Louis, Yoff, Hann, Mbour, Joal and Kafountine	03–49	88,947	TL	19.2*	48	1.5	2004–2019
	Gmelin, 1789									
										(16 years)
	(Chub mackerel)									
Mugilidae	*Mugil cephalus*	Northern Area (from 16° 04’ N to 14° 36’ N) and Southern Area (from 14° 36’ N to 13° 36’ N and from 13° 04’ N to 12°20’ N)	Kayar, Saint-Louis, Yoff, Hann, Mbour, Joal and Kafountine	03–70	94,875	TL	50.7**	66	1.5	2005–2019
	Linnaeus, 1758									(15 years)
	(Flathead grey mullet)									
Alosinae	*Sardina pilchardus*	Northern Area (from 16° 04’ N to 14° 36’ N)	Kayar, Saint-Louis and Yoff	03–31	7,856	TL	16.5***	30	1.5	2005–2019
	Walbaum, 1792									
										(15 years)
	(European pilchard)									
Dorosomatinae	*Ethmalosa fimbriata* Bowdich, 1825	Southern Area (from 14° 36’ N to 13° 36’ N and from 13° 04’ N to 12°20’ N)	Hann, Mbour, Joal and Kafountine	03–39	141,045	TL	21.5****	36.4	1.5	2004–2019
										(16 years)
	(Bonga shad)									
Caranginae	*Trachurus trecae*	Northern Area (from 16° 04’ N to 14° 36’ N) and Southern Area (from 14° 36’ N to 13° 36’ N and from 13° 04’ N to 12°20’ N)	Kayar, Saint-Louis, Yoff, Hann, Mbour, Joal and Kafountine	03–39	74,216	TL	18.7*****	35.6	1.5	2004–2019
	Cadenat, 1950									
										(16 years)
	(Cunene horse mackerel)									

TL: Total Length (cm); N: Number of individual; *L*_*50*_ (cm) represents the size at which 50% of individuals in a given population reach the maturity; *L*_*∞*_: L_t_ value when the growth rate is zero; *M/K* = 1.5 is proposed as an evolutionary ratio.

*[53]

**[54]

***[55]

****[1]

*****[56].

### 2.3. Data sets

The equations used in this study have been described by Froese et al. [11]. The calculations were performed mainly with the Gibbs Bayesian sampler software JAGS [35], R-code used for adding the random noise and for the analysis [11], and its execution using the statistical language R [36]. The simulation carried out in this work involves the empirical estimation of the stock status (*i*.*e*., *Z/K*, *B/B*_*MSY*_, *B/B*_*0*_) as well as catch control indicators (*L*_*c*_, *L*_*opt*_ and *L*_*c_opt*_) in order to adjust the fishing effort to sustainable stock exploitation of five species of small fish were estimated from monthly LF data (Table 1, Supplementary material A in S1 File). Using the *B/B*_*0*_, *B/B*_*msy*_, *L*_*c*_*/L*_*m*_ and *L*_*mean*_*/L*_*opt*_ indicators, the status of a stock can be defined [11, 16, 37] as: "healthy" if *B/B*_*msy*_ ≧ 1, "slightly overfished" if 0.8 ≦ *B/B*_*msy*_ < 1, "overfished" if 0.5 ≦ *B/B*_*msy*_ < 0.8, "grossly overfished" if 0.2 ≦ *B/B*_*msy*_ < 0.5, "collapsed" if *B/B*_*msy*_ < 0.2 and if *B/B*_*0*_ (0.4–0.5) as the reference limit of the biomass of a stock.

Length-based Bayesian biomass (LBB) model of Froese et al. [11] included in ‘TropFishR’ (Tropical Fisheries Analysis with R) package [38] was also used in this study. The use of ’TropFishR’ will allow a good estimation of *L*_*∞*_ (Table 1) and will be used in the LBB model to reduce the uncertainties in the results [11]. These software packages contain many promising new features, but still include the Powell-Wetherall (P–W) method [39] as a central component of the proposed analyses [38]. The P—W method allows to estimate *L*_*∞*_ from a linearized transformation of the annual length frequency data (LFD) (*i*.*e*., the "catch curve"). To do this, the mean lengths (*L*_*mea*n_) of all fish larger than the catch length (*L*_*c*_) are calculated. This curve is plotted from a regression analysis of the results obtained (*L_mean_*−*L_c_*) using a simple linear function of the form *L*_∞_ = *a*/−*b*. A knife-edge selection (Eq. 8 and 10; S1 File) can lead to an overestimation of the yield per recruit (e.g. short-lived species) when the selection ogive overlaps [40, 41]. A length group approach can avoid this bias. Indeed, by calculating the yield per recruit separately for each length group, it can avoid overestimating the latter [11]. Thus, it allows estimating *M/K*, *L*_*∞*_, and *CV*_*L∞*_ from the available data. The LBB method also assumes fluctuations in growth, mortality and recruitment around the mean values over the age range of the LF sample [11]. Indeed, due to lack of additional information, length-based methods cannot determine whether the observed difference in the frequency of many small and a few large individuals is caused by an unusually strong cohort of recruits or a strong removal of large fish [12]. Therefore, if this assumption is violated, it may lead to multiple peaks and biases in the results obtained [11]. The *M/K* ratio (Table 1) will be used in the LBB model equations. This estimate of *M/K* at a value of 1.5 corresponds to the peak of reproduction of the cohort relative to the average peak of the somatic growth rate of the cohort [19]. Length-at-first maturity data (*L*_*50*_) and life cycle parameters for LBB model were obtained from literature (see Table 1). The equations and assumptions underlying the results and conclusions of this study are presented in Supplementary material B in S1 File [5, 15, 19, 42].

## 3. Results

**Fig 3:**
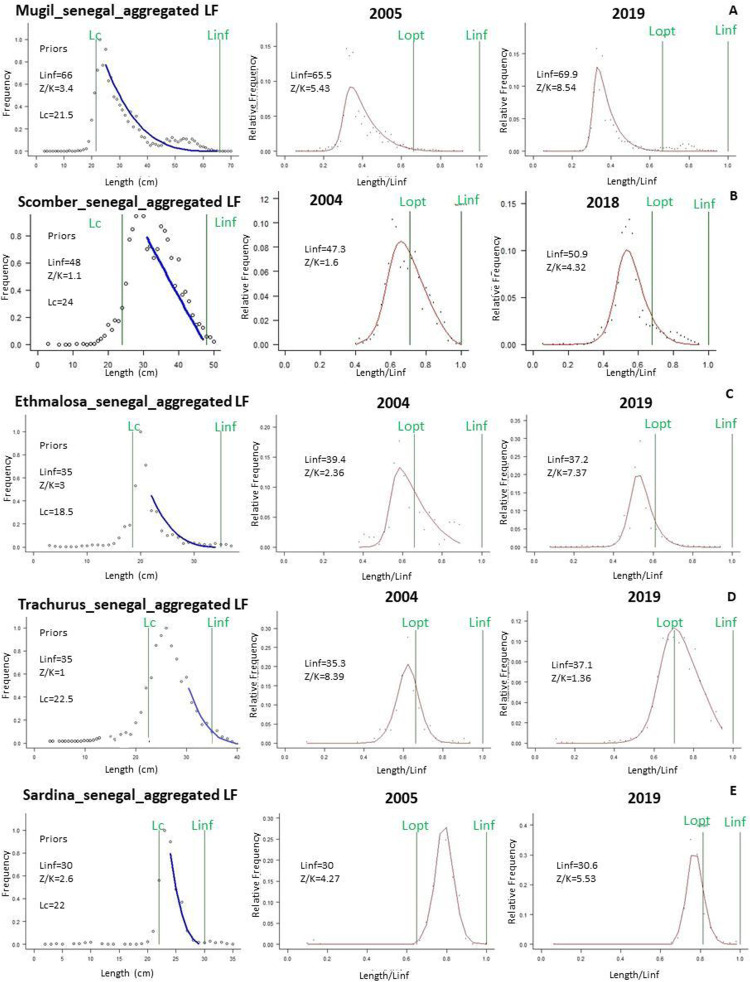
Graphical outputs of LBB analyses, showing the fit of the main LBB equation (see Table 2). The blue line (left) represents the fit of the data and the LBB estimation, and the red line (right) is the evaluation from the LBB method of stock resources. Fitness to the fully selected part of the catch in the numbers curve used to obtain *L*_*inf*_ (cm), *L*_*c*_ (cm), and *Z/K* priors for *Mugil cephalus* (A), *Scomber colias* (B), *Ethmalosa fimbriata* (C), *Trachurus trecae* (D), and *Sardina pilchardus* (E). Black dots indicated the observed LF data (total length). *L*_*opt*_ and *L*_*inf*_ were illustrated by green line.

The stock status indicators of the five species studied obtained from the LBB analysis varies between species (Fig 3, Table 2 and Supplementary material D in S1 File). Fig 3 shows the results of the specific evaluation of the LBB method. The blue line in the figures represents the fit of the data and the estimation of the LBB. The LBB model gives a good fit and shows the accumulated LF data used to estimate the priors (Fig 3). Indeed, all LF data showed good patterns to reflect the resource condition and met the requirements of the LBB (Fig 3). The curve points shows the fit for each stock, which allows the estimation of the fishery reference points, the number of years fished, i.e. *M/K*, *F/M*, *B/B*_*MSY*_, *B/B*_*0*_, *L*_*mean*_*/L*_*opt*_ and *L*_*c*_*/L*_*c_opt*_. *L*_*opt*_ indicate a relatively good stock condition. The values of *F/K*, *B/B*_*0*_, *B/B*_*MSY*_ presented by the LBB model for the 5 species vary from 1.5 to 10, 0.047 to 0.52 and 0.12 to 1.4 respectively (Table 2). The *L*_*∞*_, *L*_*c*_ and *Z/K* priors ranged from 30.5 to 69.8 cm, 18.5 and 29.8 cm and 3 to 12 respectively (Fig 3 and Table 2).

**Table 2:** Fishery statuses of the five pelagic species assessed in Senegalese waters presented by LBB [*L*_*∞*_, *L*_*c50*_, *L*_*c*_*/ L*_*∞*_, *F/M*, *F/K*, *Z/K*, *B/B*_*0*_ and *B/B*_*MSY*_ and their respective 95% confidence intervals (numbers in brackets)].

Species	*L*_*∞*_ (cm)	*L*_*c50*_ (cm)	*L*_*c*_ */ L*_*∞*_	*L*_*c*_ */ L*_*c_opt*_	*L*_*95th*_ */*	*L*_*mean*_ */*	*F/M*	*F/K*	*Z/K*	*B/B*_*0*_	*B/B*_*MSY*_	Stock status
					*L*_*∞*_	*L*_*opt*_						
*Scomber colias*	50.8	30.6	0.6	1.2	0.94	1.1	0.75 (0.52–1)	1.1	2.7	0.64	1.7	Healthy
	(50.2–51.4)	(30.2–30.9)	(0.59–0.61)					(0.86–1.3)	(2.5–2.9)	(0.12–1.4)	(0.32–3.7)	
*Mugil cephalus*	69.8	21.2	0.3	0.5	0.94	0.61	4.3	6.6	8.1	0.047	0.13	Collapsed
	(69–70.9)	(21.2–21.3)	(0.3–0.3)				(3.5–5.4)	(6.1–7)	(7.8–8.4)	(0.035–0.062)	(0.094–0.17)	
*Sardina pilchardus*	30.5	18.5	0.61	1	0.78	0.82	13	10	11	0.04	0.1	Collapsed
	(30.2–31)	(18.3–18.6)	(0.61–0.62)				(-30-43)	(9–12)	(9.9–12)	(-0.055–0.12)	(-0.14–0.3)	
*Ethmalosa fimbriata*	37.2	19.1	0.52	0.57	0.95	0.92	5.8	10	12	0.084	0.24	Grossly overfished
	(36.7–37.8)	(19–19.2)	(0.52–0.52)				(4.7–7)	(9.5–11)	(11–13)	(0.064–0.11)	(0.18–0.31)	
*Trachurus trecae*	37.1	24.4	0.65	1.2	0.93	1.1	1	1.5	3	0.52	1.4	Healthy
	(36.6–37.5)	(24.2–24.6)	(0.65–0.65)									
							(0.86–1.3)	(1.3–1.8)	(2.8–3.2)	(0.28–0.91)	(0.77–2.5)	

*L*_*∞*_: L_t_ value when the growth rate is zero; *L*_*c50*_: length at first capture *L*_*c*_ where 50% of the individuals are retained by the gear; *L*_*c*_*/ L*_*∞*_: relative length at first capture; *L*_*95th*_*/L*_*∞*_ is the 95th percentile length to asymptotic length; *L*_*mean*_*/L*_*opt*_: current size and age composition of health state; *F/M*: relative fishing mortality; *F/K*: fishing mortality rate (*F*) to somatic growth rate; Z*/K* is the ratio of the total mortality rate *Z* to somatic growth rate; *B/B*_*0*_: current biomass relative to unfished biomass; *B/B*_*MSY*_: the ratio of observed biomass, *B*, to the biomass that would provide maximum sustainable yield, *B*_*MSY*_.

The simulations showed that *M*. *cephalus* (*F/M* = 4.3, *B/B*_*0*_ = 0.047 and *B/B*_*MSY*_ = 0.13) and *S*. *pilchardus* (*F/M* = 13, *B/B*_*0*_ = 0.04 and *B/B*_*MSY*_ = 0.1) are in a collapsed state. *Ethmalosa fimbriata* (*F/M* = 5.8, *B/B*_*0*_ = 0.013 and *B/B*_*MSY*_ = 0.24) is overexploited. While the stock of *Scomber colias* (*F/M* = 0.75, *B/B*_*0*_ = 0.64 and *B/B*_*MSY*_ = 1.7) and *T*. *trecae* (*F/M* = 1, *B/B*_*0*_ = 0.52 and *B/B*_*MSY*_ = 1.4) are healthy (Table 2).

The values obtained for *L*_*c*_*/L*_*c_opt*_ of *M*. *cephalus* (*L*_*c*_*/L*_*c_opt*_ = 0.5) and *E*. *fimbriata* (*L*_*c*_*/L*_*c_opt*_ = 0.57) are below unity (1). This means a truncated length structure and the fishing of small specimens. However, for *S*. *pilchardus* (*L*_*c*_*/L*_*c_opt*_ = 0.59) and *T*. *trecae and S*. *colias* (*L*_*c*_*/L*_*c_opt*_ = 1.2 respectively), the *L*_*c*_*/L*_*c_opt*_ ≥ 1 suggesting the presence of larger specimens (Fig 3 and Table 2).

## 4. Discussion

The study of stock status indicators of these five small pelagic species, which are species shared with neighbouring countries, cannot be isolated and any exploitation outside the maritime borders of Senegal has a significant impact on the evolution of these parameters in Senegalese waters [5]. In migratory species, such as these small pelagics, input data for growth studies are often biased (some elements may be missing, as the entire cohort is not present in the area where the samples are taken). Furthermore, representative samples of all class sizes might not be obtained in other regions of northwest Africa regions, because marginal stocks might be absent or under-sampled [43].

The results of the LBB model confirmed high fishing effort for all stocks with values of *F/M* ≥ 1, *F/K* ≥ 3, and high values of *Z/K*, which are indicators of intensive fishing (Table 2). The stock status indicator (*B/B*_*MSY*_) produced by the LBB model showed that the stocks of *M*. *cephalus* and *S*. *pilchardus* are collapsed, while the stocks of *E*. *fimbriata* isoverfished. The results obtained for *T*. *trecae* and *S*. *colias* indicate that the stock is in good condition. These results differ little from those obtained by the Fishery Committee for the Eastern Central Atlantic (CECAF) during the last sub-regional assessment [44]. According to the results obtained by CECAF [44], *T*. *trecae* and *E*. *fimbriata* are overexploited while *S*. *pilchardus* and *S*. *colias* are not exploited to the full and fully exploited, respectively. This difference in results may be due to the assessment method used or the migratory ranges of the species. However, no assessment method had been applied to *M*. *cephalus* on a national or sub-regional level.

The regional assessments carried out in the framework of the CECAF working groups combine length frequency data from several countries (e.g. Gambia, Mauritania, Senegal, Morocco), using length-cohort analysis (LCA) [44]. This method is used to determine the long-term effects on yield per recruit of changes in fishing effort and/or mesh size and various indices of fishing effort at length such as *F*, *F/Z* [45]. However, it has some limitations. Indeed, the LCA requires estimates or assumptions on the underlying growth rates of the fish concerned assuming that the population is in equilibrium [45]. As a result, the choice of input growth parameters can critically influence the results obtained. High individual growth variation and poor growth data can also bias the results produced (e.g. *L*_*∞*_, *K*, *F*) [46]. It should be noted that most of the assessments used for these species by CECAF (e.g. LCA) do not provide estimates of *B/B*_*0*_ [45]. However, the LBB model allows, with a minimum of data, an estimate of the current exploited biomass relative to the unexploited biomass (*B/B*_*0*_) as well as the relative fishing mortality *F/M*. Using LF samples representative of the main gear used or even the main landing sites of the species may be sufficient to describe the biological parameters and get an idea of the status of some stocks considered data poor [11]. In order to reduce uncertainties, this model allows an estimation of *B/B*_*0*_ by including the "true" values within their 95% confidence limits. Froese et al. [11] also demonstrated that the LBB model can be used in fisheries using other types of fishing gear such as gillnets; the main fishing gear in Senegal’s artisanal fishery that targets small pelagics [21]. However, as a limitation, the LBB model only performs well if the LFs are representative of the length composition of the exploited phase of the stock or if the selectivity is different from the logistic form [47]. High interannual variability in recruitment can lead to multiple peaks and poor analytical results. Indeed, without additional information, length-based methods cannot determine whether the observed difference in the frequency of many small and a few large individuals is caused by an unusually strong cohort of recruits or by a strong removal of large fish [12]. Other indicators obtained from the LBB model can also provide insight into the status of the stock from the LFs [11, 19]. Indeed, the results of this study showed differences in the stocks according to the length indicators (e.g., *L*_*mean*_*/L*_*opt*_, *L*_*c*_*/L*_*c_opt*_, *L*_*95th*_*/L*_*∞*_; Table 2). The results of *L*_*mean*_*/L*_*opt*_ ratios were ≤ 1 for 3 species (*S*. *pilchardus*, *E*. *fimbriata* and *M*. *cephalus*), suggesting a truncated length structure. The *L*_*c*_*/L*_*c_opt*_ indicator was also ≤ 1 for *E*. *fimbriata* and *M*. *cephalus*, suggesting that the individuals caught for these species were too small. Similarly, the estimated *L*_*95th*_*/L*_*∞*_ ratio was close to unity (> 0.9) for four of the five species (*T*. *trecae*, *E*. *fimbriata*, *S*. *colias* and *M*. *cephalus*). This suggests the presence of at least some large fish in these stocks. These results could be related to the migratory behavior, variability of environmental conditions, the socio-economic importance of these stocks shared from one border country to another or the size of the individuals caught. Indeed, given the economic and social importance of artisanal pelagic fishing, Senegalese law provides it with a certain form of protection, partially protecting it from competition from industrial fishing [2]. As a result, there is a certain amount of pressure on the resources exploited. Thus, we note that the average size of individuals caught in the species studied is below the *L*_*50*_ (Table 1). In the long term, this fact could prevent the sustainability of small pelagic stocks because it does not allow them to renew themselves properly. Indeed, sardinella species show spatial and temporal distribution patterns by size group [4, 5]. Indicators based on length frequencies can be used to establish fisheries management measures [19, 48]. Fishing at *L*_*c*_ (see S1 File) would allow all fish to spawn at least once before being caught. This will help to replenish and maintain healthy spawning stocks. However, such a tactic is only possible if recruitment is successful each year. Therefore, the environment will have to be taken into account in this sustainable fishing hypothesis. The second indicator *L*_*c_opt*_ (20, 21, 34, 21 and 47 cm for *S*. *pilchardus*, *E*. *fimbriata*, *S*. *colias*, *T*. *trecae* and *M*. *cephalus*, respectively) is the length where the maximum yield can be obtained. This focus on large specimens (*L*_*c_opt*_ > *L*_*50*_) is based on growing evidence that older fish play several important roles in the long-term survival of a population (e.g. egg production; [49]). Thus, the latter indicator *L*_*c_opt*_ would allow for a fishing strategy that does not catch any mega-parent fish [48]. According to Froese et al. [19], to achieve maximum yield for a given *F*, *L*_*c*_ can be increased to allow for a longer unexploited growth phase, until the exploitable biomass and thus the catch per unit effort (CPUE) reaches a maximum. Thus, any catch at *L*_*c_opt*_, which is close to *L*_*c*_ and therefore higher than *L*_*50*_, could better achieve sustainability.

However, given the exploitative status of these species, several management measures have been implemented in Senegal. These management measures have been introduced by both the government and the local co-management committees [2]. They include a ban on certain non-selective gear such as nylon or monofilament nets. These types of nets are non-biodegradable and are often lost on fishing grounds near wrecks and rocky bottoms (protection, reproduction), where the concentration of fish is high. These lost nets continue to fish unnecessarily for decades (ghost fishing) [50]. Management measures also include mesh sizes and minimum catch sizes. Controlling minimum catch sizes in fishing areas is also an essential tool for regulating fishing effort. Article 39 of the draft decree implementing the Maritime Fisheries Code prohibits the capture, transport and sale of fish below the size and weight limits. There is as yet no government restrictions on the number of pirogues or on the quantity of fish landed. Also noteworthy are the creation of Marine Protected Areas (MPAs), no-fishing zones and the dumping of artificial reefs [2]. All these measures and management tools contribute to the preservation of biodiversity, the maintenance of essential ecological processes (e.g. enrichment of the coastal marine environment by mangroves), the protection of a natural wonder, the conservation of important environments for living beings in the sea, the preservation of threatened species, the safeguarding of cultural and historical values, etc [2]. Given the level of exploitation of these species, the proposals for management plans which take time to be validated and the development and modernisation of artisanal fishing, one can only be pessimistic with regard to management that is intended to be sustainable. Therefore, in order to preserve the reproductive potential of the entire stock of these species, we also recommend (1) maintain and apply the current regulations on mesh size, (2) resort to a drastic reduction in fishing effort, (3) penalise negatively, prior to a good awareness-information campaign, the capture, sale and processing of juveniles and (4) reduce the number of purse seiners (ST) in activity, as these fleets operate with a type of non-selective gear that has a high potential for by-catch. The Senegalese Fishing Code of 2015 (Law No. 2015–18 of 13 July 2015, Art. 38) clearly states that the capture, transport, transfer, holding, sale, for sale and purchase of several fish species including small pelagic fish ≤ 18 cm is prohibited. However, despite its legal prohibition, there has been a strong presence of juvenile coastal small pelagic fish in landings in recent years [51]. Due to their strong presence near the Senegalese coast, these juveniles are more accessible to artisanal fishing in terms of effort, energy and fishing time [51]. These small individuals are often used in processed products, as raw materials for the manufacture of fishmeal, fish oil [52] or as bait-by-bait boats (direct or indirect fishing). There are no government restrictions yet on the number of boats or the number of fish landed. The socio-economic consequences of these types of exploitation of small pelagics therefore raise the question of the medium and long-term economic viability of the fisheries, which depends on the variability of the resources exploited.

## 5. Conclusion

Given the shared nature of small pelagic fish, concerted management between the countries concerned is therefore more effective. The desired concertation should focus on the fishing possibilities of each country and the harmonisation of technical management measures. However, the strategy to be developed to achieve effective application of these measures may differ from one country to another depending on the specificities of the fishery through existing sub-regional organisations such as the SRFC. This study allowed stock assessments to be carried out for important small pelagic fisheries on the basis of representative lengths collected in Senegalese waters. The results showed that the LBB method only requires LF data to assess fishery resources in data-poor fisheries at different temporal and spatial scales, which can be useful when formulating scientific advice. Indeed, four out of five of the stocks studied are collapsed (*M*. *cephalus* and *S*. *pilchardus*) or overexploited (*E*. *fimbriata*). Only *S*. *colias* and *T*. *trecae* show a stock in good condition. In order to contribute to the recovery of overexploited or collapsed stocks, management authorities should use the *L*_*c_opt*_ management indicator to set species-specific size limits and ensure that these limits are enforced. Indeed, catching individuals at *L*_*c_opt*_, 25, 21, 43 and 18 cm for *S*. *colias*, *E*. *fimbriata*, *M*. *cephalus* and *S*. *pilchardus*, respectively, should be a natural guarantee against recruitment failure and allow individuals to ensure the long-term survival of populations, in a context of data poor fisheries. However, there are not many studies based on length frequencies in West Africa. Therefore, the results need to be verified in combination and compared with similar approaches to be more convincing and acceptable for these types of fisheries.

## Supporting information

S1 File (DOCX)
