# Supplementary material for: Length-based assessment of five small pelagic fishes in the Senegalese artisanal fisheries
Source: PLoS One. 2022 Dec 30;17(12):e0279768. doi: 10.1371/journal.pone.0279768 (PMC9803127; doi:10.1371/journal.pone.0279768)

**Supplementary material A:** Length frequencies of the five pelagic fish species used to run the LBB models.

| *Sardina pilchardus* (2005-2019) | | | | | | | | | | | | |
| --- | --- | --- | --- | --- | --- | --- | --- | --- | --- | --- | --- | --- |
| Length/Month | 1 | 2 | 3 | 4 | 5 | 6 | 7 | 8 | 10 | 11 | 12 | Total |
| 3 | 3 | 0 | 1 | 0 | 0 | 1 | 0 | 0 | 0 | 0 | 0 | 5 |
| 4 | 12 | 0 | 4 | 0 | 0 | 0 | 0 | 0 | 0 | 0 | 0 | 16 |
| 5 | 1 | 0 | 12 | 0 | 0 | 0 | 0 | 0 | 0 | 0 | 0 | 13 |
| 6 | 0 | 0 | 0 | 0 | 0 | 0 | 0 | 0 | 0 | 0 | 1 | 1 |
| 7 | 0 | 0 | 0 | 0 | 0 | 0 | 0 | 1 | 0 | 0 | 0 | 1 |
| 8 | 0 | 0 | 0 | 0 | 0 | 0 | 0 | 1 | 0 | 0 | 0 | 1 |
| 9 | 0 | 0 | 0 | 0 | 0 | 0 | 0 | 2 | 0 | 0 | 0 | 2 |
| 10 | 0 | 0 | 0 | 0 | 0 | 0 | 0 | 3 | 0 | 0 | 0 | 3 |
| 11 | 0 | 0 | 0 | 1 | 0 | 0 | 0 | 0 | 0 | 0 | 0 | 1 |
| 12 | 0 | 0 | 0 | 1 | 0 | 0 | 0 | 1 | 0 | 0 | 0 | 2 |
| 13 | 0 | 0 | 1 | 1 | 0 | 0 | 0 | 0 | 0 | 0 | 0 | 2 |
| 14 | 1 | 0 | 0 | 2 | 0 | 0 | 0 | 0 | 0 | 0 | 0 | 3 |
| 15 | 0 | 0 | 0 | 1 | 0 | 0 | 0 | 0 | 0 | 0 | 0 | 1 |
| 16 | 0 | 0 | 1 | 2 | 0 | 0 | 0 | 0 | 0 | 0 | 0 | 3 |
| 17 | 0 | 0 | 2 | 0 | 0 | 0 | 0 | 0 | 0 | 0 | 0 | 2 |
| 18 | 0 | 0 | 5 | 0 | 0 | 0 | 0 | 0 | 0 | 0 | 0 | 5 |
| 19 | 3 | 0 | 4 | 0 | 1 | 0 | 0 | 0 | 0 | 0 | 0 | 8 |
| 20 | 30 | 9 | 7 | 0 | 2 | 3 | 0 | 0 | 0 | 1 | 4 | 56 |
| 21 | 181 | 57 | 52 | 4 | 6 | 2 | 0 | 0 | 0 | 8 | 60 | 370 |
| 22 | 536 | 212 | 237 | 60 | 56 | 10 | 0 | 0 | 0 | 59 | 154 | 1324 |
| 23 | 842 | 440 | 289 | 77 | 145 | 37 | 0 | 0 | 0 | 127 | 253 | 2210 |
| 24 | 647 | 399 | 219 | 57 | 96 | 63 | 1 | 0 | 0 | 117 | 224 | 1823 |
| 25 | 301 | 253 | 95 | 24 | 39 | 43 | 2 | 0 | 1 | 30 | 121 | 909 |
| 26 | 147 | 192 | 63 | 23 | 13 | 55 | 5 | 0 | 1 | 9 | 198 | 706 |
| 27 | 41 | 35 | 28 | 9 | 10 | 19 | 3 | 0 | 0 | 2 | 50 | 197 |
| 28 | 9 | 4 | 4 | 5 | 3 | 2 | 2 | 0 | 0 | 3 | 33 | 65 |
| 29 | 2 | 1 | 4 | 2 | 2 | 3 | 1 | 0 | 0 | 1 | 11 | 27 |
| 30 | 2 | 0 | 1 | 2 | 4 | 7 | 3 | 0 | 0 | 2 | 4 | 25 |
| 31 | 2 | 0 | 2 |  | 2 | 5 | 1 | 0 | 0 |  | 16 | 28 |
| Total | 2760 | 1602 | 1031 | 271 | 379 | 250 | 18 | 8 | 2 | 359 | 1129 | 7809 |

| *Ethmalosa fimbriata* (2004-2019) | | | | | | | | | | | | | |
| --- | --- | --- | --- | --- | --- | --- | --- | --- | --- | --- | --- | --- | --- |
| Length/Month | 1 | 2 | 3 | 4 | 5 | 6 | 7 | 8 | 9 | 10 | 11 | 12 | Total |
| 3 |  | 1 | 1 | 2 |  |  |  | 3 | 1 |  | 1 |  | 9 |
| 4 | 28 | 18 | 47 | 19 | 12 | 5 | 6 | 10 | 12 | 34 | 21 | 12 | 224 |
| 5 | 2 |  | 5 | 2 | 1 | 5 |  | 1 |  |  | 1 |  | 17 |
| 6 | 1 | 1 | 6 | 1 | 3 | 2 | 6 |  | 1 | 1 |  |  | 22 |
| 7 |  | 2 | 35 | 2 | 6 | 2 | 4 |  |  |  |  | 2 | 53 |
| 8 |  | 6 | 12 | 19 | 30 | 7 | 1 |  | 2 | 1 |  |  | 78 |
| 9 | 2 | 10 | 70 | 108 | 75 | 31 | 7 | 1 | 5 | 7 | 3 |  | 319 |
| 10 | 10 | 29 | 45 | 81 | 102 | 43 | 10 | 4 | 6 | 4 | 1 | 1 | 336 |
| 11 | 15 | 23 | 89 | 164 | 103 | 65 | 26 | 11 | 8 | 23 | 5 | 19 | 551 |
| 12 | 26 | 26 | 106 | 148 | 132 | 71 | 41 | 26 | 13 | 29 | 8 | 33 | 659 |
| 13 | 52 | 35 | 69 | 21 | 50 | 35 | 18 | 13 | 16 | 74 | 22 | 45 | 450 |
| 14 | 60 | 42 | 69 | 90 | 82 | 65 | 39 | 17 | 18 | 75 | 30 | 47 | 634 |
| 15 | 69 | 59 | 122 | 86 | 160 | 118 | 59 | 38 | 36 | 64 | 43 | 45 | 899 |
| 16 | 170 | 154 | 325 | 351 | 436 | 294 | 189 | 189 | 163 | 135 | 107 | 69 | 2582 |
| 17 | 511 | 525 | 724 | 728 | 927 | 753 | 586 | 543 | 558 | 397 | 404 | 299 | 6955 |
| 18 | 390 | 420 | 584 | 524 | 578 | 650 | 613 | 620 | 563 | 413 | 298 | 308 | 5961 |
| 19 | 1388 | 1648 | 1948 | 1931 | 2023 | 1938 | 1958 | 1939 | 1605 | 991 | 991 | 904 | 19264 |
| 20 | 2653 | 3344 | 3721 | 3303 | 2783 | 3223 | 3665 | 3958 | 3146 | 1502 | 1671 | 1858 | 34827 |
| 21 | 1601 | 2455 | 2594 | 2393 | 1884 | 1863 | 2091 | 2856 | 1609 | 669 | 651 | 1114 | 21780 |
| 22 | 674 | 1084 | 1239 | 875 | 600 | 872 | 1086 | 1052 | 463 | 184 | 193 | 453 | 8775 |
| 23 | 684 | 1317 | 1401 | 1124 | 895 | 761 | 846 | 1070 | 327 | 210 | 196 | 363 | 9194 |
| 24 | 366 | 642 | 661 | 440 | 292 | 416 | 418 | 491 | 166 | 139 | 97 | 228 | 4356 |
| 25 | 346 | 635 | 448 | 303 | 154 | 283 | 281 | 389 | 114 | 106 | 78 | 155 | 3292 |
| 26 | 400 | 594 | 754 | 460 | 365 | 180 | 356 | 282 | 121 | 167 | 72 | 142 | 3893 |
| 27 | 263 | 464 | 690 | 330 | 257 | 159 | 257 | 204 | 110 | 108 | 57 | 126 | 3025 |
| 28 | 120 | 92 | 86 | 92 | 64 | 97 | 156 | 105 | 66 | 74 | 47 | 102 | 1101 |
| 29 | 117 | 153 | 327 | 78 | 67 | 100 | 146 | 78 | 68 | 86 | 57 | 115 | 1392 |
| 30 | 146 | 150 | 108 | 102 | 81 | 102 | 138 | 68 | 71 | 86 | 60 | 183 | 1295 |
| 31 | 136 | 143 | 212 | 86 | 91 | 158 | 114 | 58 | 55 | 54 | 73 | 161 | 1341 |
| 32 | 163 | 151 | 144 | 90 | 137 | 149 | 69 | 37 | 27 | 33 | 76 | 181 | 1257 |
| 33 | 122 | 146 | 65 | 106 | 128 | 164 | 139 | 71 | 65 | 61 | 64 | 139 | 1270 |
| 34 | 124 | 138 | 209 | 122 | 166 | 212 | 130 | 86 | 43 | 97 | 124 | 190 | 1641 |
| 35 | 79 | 113 | 98 | 96 | 112 | 108 | 85 | 39 | 28 | 54 | 66 | 99 | 977 |
| 36 | 117 | 101 | 132 | 106 | 147 | 130 | 104 | 74 | 40 | 114 | 92 | 198 | 1355 |
| 37 | 41 | 50 | 51 | 55 | 74 | 51 | 36 | 28 | 12 | 56 | 53 | 99 | 606 |
| 38 | 29 | 23 | 21 | 48 | 45 | 23 | 13 | 14 | 13 | 31 | 30 | 55 | 345 |
| 39 | 35 | 26 | 30 | 31 | 31 | 30 | 11 | 15 | 6 | 39 | 26 | 30 | 310 |
| Total | 10940 | 14820 | 17248 | 14517 | 13093 | 13165 | 13704 | 14390 | 9557 | 6118 | 5718 | 7775 | 141045 |

| *Trachurus trecae* (2004-2019) | | | | | | | | | | | | | |
| --- | --- | --- | --- | --- | --- | --- | --- | --- | --- | --- | --- | --- | --- |
| Length/Month | 1 | 2 | 3 | 4 | 5 | 6 | 7 | 8 | 9 | 10 | 11 | 12 | Total |
| 3 | 0 | 2 | 2 | 3 | 0 | 0 | 0 | 0 | 0 | 0 | 0 | 1 | 8 |
| 4 | 672 | 289 | 205 | 282 | 335 | 100 | 2 | 1 | 8 | 2 | 27 | 149 | 2072 |
| 5 | 11 | 16 | 17 | 25 | 38 | 42 | 1 | 0 | 2 | 0 | 6 | 12 | 170 |
| 6 | 0 | 1 | 0 | 3 | 16 | 0 | 0 | 0 | 0 | 0 | 2 | 1 | 23 |
| 7 | 0 | 1 | 0 | 1 | 1 | 0 | 0 | 0 | 0 | 0 | 2 | 1 | 6 |
| 8 | 0 | 0 | 0 | 10 | 1 | 0 | 0 | 0 | 0 | 0 | 0 | 14 | 25 |
| 9 | 0 | 1 | 0 | 5 | 0 | 0 | 0 | 0 | 0 | 0 | 0 | 2 | 8 |
| 11 | 0 | 0 | 1 | 0 | 0 | 0 | 0 | 0 | 0 | 0 | 1 | 0 | 2 |
| 12 | 2 | 2 | 0 | 1 | 0 | 0 | 0 | 0 | 0 | 0 | 1 | 0 | 6 |
| 13 | 6 | 9 | 0 | 2 | 1 | 2 | 0 | 0 | 1 | 1 | 3 | 15 | 40 |
| 14 | 19 | 42 | 9 | 0 | 2 | 1 | 0 | 0 | 0 | 1 | 2 | 25 | 101 |
| 15 | 17 | 59 | 31 | 15 | 6 | 1 | 0 | 0 | 0 | 0 | 8 | 21 | 158 |
| 16 | 37 | 64 | 52 | 28 | 22 | 5 | 1 | 2 | 1 | 3 | 8 | 25 | 248 |
| 17 | 39 | 82 | 90 | 64 | 51 | 22 | 0 | 4 | 0 | 3 | 10 | 28 | 393 |
| 18 | 54 | 69 | 108 | 100 | 132 | 110 | 6 | 2 | 1 | 6 | 17 | 45 | 650 |
| 19 | 53 | 79 | 87 | 136 | 164 | 132 | 5 | 0 | 3 | 9 | 25 | 82 | 775 |
| 20 | 83 | 90 | 87 | 148 | 213 | 83 | 12 | 0 | 9 | 16 | 101 | 267 | 1109 |
| 21 | 112 | 67 | 74 | 181 | 183 | 65 | 8 | 0 | 3 | 19 | 93 | 409 | 1214 |
| 22 | 362 | 137 | 104 | 294 | 183 | 96 | 23 | 0 | 6 | 25 | 162 | 556 | 1948 |
| 23 | 477 | 256 | 256 | 294 | 351 | 108 | 39 | 2 | 6 | 61 | 198 | 660 | 2708 |
| 24 | 558 | 450 | 580 | 605 | 514 | 162 | 49 | 11 | 14 | 70 | 288 | 955 | 4256 |
| 25 | 635 | 589 | 825 | 924 | 731 | 154 | 50 | 21 | 19 | 108 | 452 | 1084 | 5592 |
| 26 | 814 | 765 | 900 | 1138 | 962 | 279 | 50 | 20 | 22 | 128 | 611 | 1198 | 6887 |
| 27 | 786 | 635 | 687 | 1047 | 869 | 289 | 40 | 24 | 16 | 118 | 596 | 991 | 6098 |
| 28 | 725 | 812 | 832 | 1054 | 672 | 297 | 46 | 19 | 21 | 127 | 495 | 854 | 5954 |
| 29 | 631 | 678 | 966 | 1021 | 486 | 186 | 18 | 9 | 25 | 91 | 356 | 752 | 5219 |
| 30 | 735 | 906 | 1114 | 926 | 437 | 145 | 29 | 7 | 12 | 72 | 316 | 748 | 5447 |
| 31 | 722 | 731 | 780 | 657 | 341 | 94 | 6 | 2 | 6 | 69 | 225 | 613 | 4246 |
| 32 | 803 | 665 | 771 | 526 | 290 | 94 | 12 | 4 | 3 | 48 | 158 | 507 | 3881 |
| 33 | 751 | 586 | 585 | 429 | 207 | 56 | 2 | 2 | 4 | 27 | 101 | 406 | 3156 |
| 34 | 856 | 595 | 556 | 456 | 250 | 75 | 8 | 3 | 2 | 16 | 87 | 369 | 3273 |
| 35 | 627 | 402 | 352 | 362 | 206 | 64 | 3 | 5 | 3 | 8 | 31 | 202 | 2265 |
| 36 | 697 | 408 | 339 | 329 | 233 | 89 | 1 | 5 | 1 | 5 | 46 | 192 | 2345 |
| 37 | 569 | 271 | 217 | 263 | 172 | 64 | 2 | 3 | 1 | 5 | 19 | 128 | 1714 |
| 38 | 477 | 179 | 144 | 202 | 160 | 42 | 0 | 4 | 3 | 1 | 14 | 84 | 1310 |
| 39 | 283 | 140 | 126 | 138 | 119 | 39 | 2 | 3 | 4 | 4 | 8 | 43 | 909 |
| Total | 12613 | 10078 | 10897 | 11669 | 8348 | 2896 | 415 | 153 | 196 | 1043 | 4469 | 11439 | 74216 |

| *Scomber colias* (2004-2018) | | | | | | | | | | | | | |
| --- | --- | --- | --- | --- | --- | --- | --- | --- | --- | --- | --- | --- | --- |
| Length/Month | 1 | 2 | 3 | 4 | 5 | 6 | 7 | 8 | 9 | 10 | 11 | 12 | Total |
| 3 | 2 | 0 | 0 | 2 | 1 | 0 | 0 | 0 | 0 | 0 | 1 | 1 | 7 |
| 4 | 6 | 0 | 0 | 0 | 0 | 0 | 0 | 0 | 0 | 0 | 0 | 3 | 9 |
| 6 | 0 | 0 | 0 | 1 | 0 | 0 | 0 | 0 | 0 | 0 | 0 | 2 | 3 |
| 7 | 2 | 0 | 0 | 0 | 0 | 0 | 0 | 0 | 0 | 1 | 1 | 0 | 4 |
| 8 | 1 | 0 | 1 | 0 | 1 | 0 | 0 | 1 | 0 | 0 | 2 | 2 | 8 |
| 9 | 0 | 1 | 0 | 1 | 0 | 1 | 0 | 0 | 0 | 0 | 0 | 3 | 6 |
| 10 | 1 | 1 | 1 | 0 | 0 | 0 | 0 | 0 | 0 | 0 | 0 | 0 | 3 |
| 11 | 1 | 0 | 0 | 2 | 0 | 0 | 0 | 0 | 0 | 0 | 0 | 0 | 3 |
| 12 | 0 | 0 | 0 | 1 | 0 | 0 | 0 | 0 | 0 | 0 | 0 | 0 | 1 |
| 13 | 1 | 0 | 2 | 3 | 0 | 1 | 0 | 0 | 0 | 0 | 0 | 0 | 7 |
| 14 | 0 | 3 | 5 | 3 | 2 | 1 | 0 | 0 | 0 | 0 | 1 | 1 | 16 |
| 15 | 0 | 0 | 2 | 10 | 7 | 3 | 0 | 0 | 0 | 0 | 0 | 0 | 22 |
| 16 | 2 | 0 | 2 | 9 | 4 | 4 | 1 | 0 | 0 | 0 | 3 | 1 | 26 |
| 17 | 5 | 2 | 5 | 7 | 13 | 20 | 0 | 3 | 0 | 1 | 2 | 3 | 61 |
| 18 | 32 | 0 | 8 | 10 | 12 | 25 | 2 | 9 | 0 | 0 | 5 | 4 | 107 |
| 19 | 72 | 2 | 6 | 16 | 16 | 19 | 11 | 5 | 0 | 1 | 7 | 10 | 165 |
| 20 | 117 | 31 | 18 | 36 | 49 | 57 | 26 | 7 | 0 | 10 | 13 | 71 | 435 |
| 21 | 45 | 26 | 32 | 24 | 46 | 29 | 37 | 7 | 6 | 16 | 18 | 103 | 389 |
| 22 | 70 | 68 | 20 | 46 | 67 | 105 | 81 | 18 | 16 | 24 | 40 | 193 | 748 |
| 23 | 84 | 98 | 66 | 65 | 83 | 113 | 79 | 26 | 28 | 58 | 80 | 203 | 983 |
| 24 | 157 | 153 | 127 | 66 | 90 | 107 | 83 | 34 | 17 | 70 | 120 | 391 | 1415 |
| 25 | 335 | 199 | 186 | 189 | 153 | 106 | 81 | 27 | 38 | 66 | 232 | 719 | 2331 |
| 26 | 512 | 287 | 251 | 359 | 320 | 158 | 82 | 58 | 35 | 73 | 374 | 924 | 3433 |
| 27 | 477 | 274 | 274 | 567 | 516 | 200 | 83 | 131 | 121 | 97 | 482 | 908 | 4130 |
| 28 | 610 | 341 | 273 | 812 | 683 | 321 | 83 | 201 | 186 | 143 | 666 | 909 | 5228 |
| 29 | 672 | 450 | 380 | 842 | 866 | 424 | 79 | 208 | 222 | 202 | 687 | 825 | 5857 |
| 30 | 708 | 465 | 345 | 384 | 615 | 568 | 73 | 45 | 66 | 154 | 531 | 696 | 4650 |
| 31 | 580 | 338 | 322 | 348 | 405 | 471 | 42 | 44 | 40 | 126 | 317 | 452 | 3485 |
| 32 | 681 | 429 | 476 | 609 | 724 | 602 | 66 | 109 | 141 | 236 | 361 | 565 | 4999 |
| 33 | 543 | 391 | 513 | 464 | 610 | 529 | 48 | 69 | 93 | 188 | 228 | 403 | 4079 |
| 34 | 531 | 341 | 474 | 482 | 509 | 305 | 35 | 64 | 117 | 157 | 231 | 541 | 3787 |
| 35 | 678 | 432 | 470 | 345 | 409 | 174 | 16 | 42 | 65 | 85 | 191 | 533 | 3440 |
| 36 | 966 | 608 | 581 | 384 | 357 | 110 | 13 | 18 | 23 | 36 | 175 | 922 | 4193 |
| 37 | 1172 | 698 | 563 | 417 | 274 | 64 | 8 | 12 | 15 | 22 | 100 | 859 | 4204 |
| 38 | 1354 | 746 | 559 | 536 | 214 | 59 | 8 | 6 | 5 | 9 | 71 | 1110 | 4677 |
| 39 | 1415 | 691 | 416 | 545 | 180 | 53 | 7 | 4 | 1 | 3 | 50 | 930 | 4295 |
| 40 | 1983 | 746 | 446 | 566 | 244 | 21 | 2 | 6 | 3 | 3 | 37 | 939 | 4996 |
| 41 | 1549 | 514 | 263 | 372 | 135 | 28 | 1 | 0 | 1 | 2 | 26 | 746 | 3637 |
| 42 | 1770 | 604 | 311 | 353 | 195 | 24 | 4 | 2 | 0 | 0 | 36 | 959 | 4258 |
| 43 | 1218 | 382 | 247 | 298 | 132 | 9 | 2 | 2 | 1 | 0 | 19 | 583 | 2893 |
| 44 | 893 | 378 | 209 | 229 | 137 | 3 | 3 | 2 | 1 | 0 | 14 | 490 | 2359 |
| 45 | 588 | 189 | 114 | 114 | 67 | 5 | 2 | 2 | 0 | 0 | 12 | 255 | 1348 |
| 46 | 301 | 136 | 95 | 107 | 78 | 4 | 2 | 2 | 2 | 0 | 8 | 176 | 911 |
| 47 | 149 | 84 | 55 | 101 | 61 | 1 | 1 | 0 | 1 | 1 | 11 | 92 | 557 |
| 48 | 72 | 56 | 18 | 67 | 31 | 2 | 0 | 4 | 0 | 0 | 4 | 55 | 309 |
| 49 | 56 | 37 | 16 | 39 | 14 | 2 | 1 | 2 | 0 | 0 | 1 | 37 | 205 |
| Total | 20411 | 10201 | 8152 | 9831 | 8320 | 4728 | 1062 | 1170 | 1244 | 1784 | 5157 | 16619 | 88679 |

| *Mugil cephalus* (2004-2019) | | | | | | | | | | | | | |
| --- | --- | --- | --- | --- | --- | --- | --- | --- | --- | --- | --- | --- | --- |
| Length/Month | 1 | 2 | 3 | 4 | 5 | 6 | 7 | 8 | 9 | 10 | 11 | 12 | Total |
| 3 | 0 | 1 | 1 | 0 | 0 | 1 | 0 | 0 | 0 | 0 | 0 | 0 | 3 |
| 4 | 0 | 0 | 0 | 0 | 0 | 1 | 0 | 1 | 0 | 0 | 1 | 1 | 4 |
| 5 | 0 | 2 | 1 | 0 | 0 | 1 | 0 | 0 | 2 | 0 | 0 | 0 | 6 |
| 6 | 1 | 0 | 0 | 0 | 0 | 0 | 0 | 0 | 0 | 0 | 0 | 1 | 2 |
| 7 | 0 | 0 | 0 | 0 | 0 | 0 | 0 | 0 | 1 | 0 | 0 | 0 | 1 |
| 8 | 1 | 0 | 0 | 0 | 2 | 1 | 2 | 0 | 0 | 0 | 0 | 2 | 8 |
| 9 | 7 | 3 | 1 | 0 | 1 | 0 | 2 | 0 | 2 | 0 | 2 | 4 | 22 |
| 10 | 1 | 0 | 0 | 2 | 0 | 0 | 0 | 0 | 2 | 0 | 0 | 0 | 5 |
| 11 | 0 | 0 | 0 | 1 | 1 | 3 | 0 | 0 | 1 | 0 | 0 | 0 | 6 |
| 12 | 0 | 0 | 0 | 3 | 7 | 3 | 1 | 1 | 1 | 0 | 1 | 1 | 18 |
| 13 | 1 | 0 | 0 | 0 | 3 | 0 | 1 | 0 | 0 | 0 | 0 | 0 | 5 |
| 14 | 0 | 0 | 0 | 2 | 4 | 2 | 3 | 0 | 0 | 0 | 0 | 0 | 11 |
| 15 | 4 | 2 | 1 | 2 | 8 | 5 | 4 | 2 | 1 | 0 | 1 | 3 | 33 |
| 16 | 5 | 10 | 0 | 1 | 7 | 7 | 2 | 9 | 2 | 0 | 2 | 5 | 50 |
| 17 | 16 | 12 | 1 | 4 | 12 | 12 | 10 | 17 | 4 | 3 | 1 | 4 | 96 |
| 18 | 22 | 9 | 12 | 7 | 8 | 31 | 18 | 20 | 28 | 21 | 15 | 6 | 197 |
| 19 | 57 | 34 | 25 | 40 | 49 | 59 | 87 | 62 | 82 | 90 | 65 | 39 | 689 |
| 20 | 159 | 94 | 104 | 226 | 197 | 186 | 246 | 226 | 189 | 306 | 143 | 146 | 2222 |
| 21 | 274 | 180 | 258 | 388 | 354 | 365 | 388 | 462 | 297 | 506 | 288 | 267 | 4027 |
| 22 | 396 | 356 | 353 | 362 | 478 | 451 | 548 | 800 | 464 | 599 | 387 | 436 | 5630 |
| 23 | 610 | 577 | 522 | 534 | 567 | 596 | 658 | 856 | 554 | 922 | 775 | 624 | 7795 |
| 24 | 467 | 493 | 501 | 546 | 590 | 495 | 441 | 636 | 345 | 787 | 578 | 458 | 6337 |
| 25 | 587 | 607 | 598 | 595 | 624 | 534 | 477 | 760 | 519 | 782 | 769 | 613 | 7465 |
| 26 | 555 | 502 | 594 | 524 | 514 | 373 | 304 | 347 | 282 | 469 | 467 | 396 | 5327 |
| 27 | 565 | 530 | 589 | 558 | 512 | 361 | 308 | 344 | 274 | 464 | 534 | 443 | 5482 |
| 28 | 467 | 421 | 456 | 372 | 369 | 262 | 208 | 243 | 213 | 336 | 406 | 384 | 4137 |
| 29 | 407 | 390 | 465 | 342 | 300 | 270 | 197 | 198 | 206 | 346 | 420 | 348 | 3889 |
| 30 | 441 | 465 | 533 | 373 | 284 | 182 | 157 | 115 | 107 | 238 | 345 | 324 | 3564 |
| 31 | 392 | 385 | 576 | 328 | 223 | 154 | 108 | 96 | 84 | 195 | 236 | 251 | 3028 |
| 32 | 487 | 350 | 450 | 228 | 170 | 126 | 105 | 82 | 57 | 176 | 261 | 251 | 2743 |
| 33 | 372 | 321 | 435 | 117 | 113 | 90 | 104 | 71 | 74 | 177 | 238 | 196 | 2308 |
| 34 | 505 | 468 | 428 | 223 | 208 | 135 | 115 | 95 | 91 | 236 | 196 | 256 | 2956 |
| 35 | 285 | 254 | 246 | 82 | 93 | 81 | 98 | 83 | 100 | 213 | 231 | 214 | 1980 |
| 36 | 563 | 462 | 361 | 205 | 174 | 89 | 89 | 74 | 112 | 177 | 137 | 242 | 2685 |
| 37 | 300 | 228 | 135 | 71 | 104 | 68 | 82 | 62 | 86 | 186 | 209 | 249 | 1780 |
| 38 | 243 | 163 | 78 | 56 | 72 | 40 | 81 | 44 | 57 | 102 | 124 | 147 | 1207 |
| 39 | 197 | 160 | 81 | 44 | 57 | 46 | 47 | 39 | 37 | 105 | 100 | 165 | 1078 |
| 40 | 145 | 103 | 56 | 44 | 44 | 30 | 31 | 42 | 49 | 64 | 84 | 108 | 800 |
| 41 | 79 | 39 | 44 | 35 | 37 | 18 | 23 | 20 | 31 | 44 | 52 | 75 | 497 |
| 42 | 118 | 99 | 67 | 39 | 63 | 18 | 27 | 17 | 26 | 31 | 64 | 81 | 650 |
| 43 | 100 | 68 | 59 | 48 | 52 | 22 | 12 | 12 | 17 | 17 | 68 | 94 | 569 |
| 44 | 82 | 46 | 87 | 40 | 44 | 17 | 8 | 8 | 7 | 19 | 48 | 48 | 454 |
| 45 | 114 | 72 | 71 | 56 | 44 | 16 | 11 | 5 | 4 | 18 | 61 | 63 | 535 |
| 46 | 148 | 116 | 76 | 85 | 59 | 22 | 13 | 2 | 5 | 22 | 75 | 163 | 786 |
| 47 | 239 | 183 | 119 | 94 | 47 | 22 | 15 | 4 | 4 | 25 | 99 | 219 | 1070 |
| 48 | 212 | 141 | 88 | 72 | 54 | 33 | 3 | 4 | 0 | 28 | 92 | 191 | 918 |
| 49 | 293 | 202 | 113 | 91 | 42 | 22 | 4 | 1 | 3 | 19 | 120 | 283 | 1193 |
| 50 | 249 | 181 | 125 | 111 | 47 | 21 | 10 | 4 | 8 | 18 | 106 | 192 | 1072 |
| 51 | 215 | 129 | 79 | 73 | 24 | 20 | 4 | 2 | 0 | 15 | 68 | 216 | 845 |
| 52 | 260 | 191 | 142 | 76 | 34 | 22 | 3 | 2 | 4 | 15 | 76 | 226 | 1051 |
| 53 | 228 | 246 | 162 | 76 | 35 | 16 | 3 | 3 | 3 | 20 | 59 | 225 | 1076 |
| 54 | 277 | 259 | 167 | 55 | 40 | 24 | 0 | 4 | 1 | 18 | 78 | 225 | 1148 |
| 55 | 200 | 159 | 170 | 64 | 27 | 10 | 0 | 2 | 2 | 8 | 61 | 120 | 823 |
| 56 | 299 | 267 | 195 | 72 | 38 | 16 | 0 | 3 | 2 | 16 | 46 | 208 | 1162 |
| 57 | 226 | 217 | 163 | 72 | 34 | 9 | 1 | 1 | 1 | 10 | 35 | 159 | 928 |
| 58 | 170 | 193 | 109 | 60 | 23 | 2 | 0 | 1 | 0 | 10 | 33 | 93 | 694 |
| 59 | 132 | 102 | 88 | 45 | 18 | 4 | 0 | 0 | 2 | 7 | 25 | 43 | 466 |
| 60 | 125 | 79 | 65 | 33 | 24 | 6 | 1 | 0 | 1 | 2 | 33 | 63 | 432 |
| 61 | 87 | 70 | 66 | 18 | 8 | 1 | 1 | 2 | 0 | 1 | 18 | 49 | 321 |
| 62 | 43 | 43 | 27 | 16 | 7 | 2 | 1 | 2 | 0 | 0 | 8 | 32 | 181 |
| 63 | 44 | 21 | 28 | 12 | 5 | 1 | 0 | 0 | 0 | 1 | 6 | 25 | 143 |
| 64 | 25 | 22 | 17 | 2 | 3 | 0 | 0 | 0 | 0 | 0 | 8 | 18 | 95 |
| 65 | 8 | 6 | 11 | 2 | 2 | 0 | 0 | 0 | 1 | 1 | 4 | 13 | 48 |
| 66 | 13 | 3 | 3 | 4 | 2 | 0 | 0 | 0 | 0 | 0 | 7 | 8 | 40 |
| 67 | 8 | 4 | 5 | 2 | 1 | 0 | 0 | 0 | 0 | 0 | 5 | 10 | 35 |
| 68 | 3 | 1 | 2 | 2 | 1 | 0 | 0 | 0 | 0 | 0 | 2 | 3 | 14 |
| 69 | 7 | 1 | 2 | 0 | 1 | 0 | 0 | 0 | 0 | 0 | 1 | 2 | 14 |
| 70 | 7 | 1 | 1 | 0 | 2 | 1 | 1 | 2 | 0 | 0 | 1 | 3 | 19 |
| Total | 12543 | 10743 | 10212 | 7635 | 6967 | 5405 | 5063 | 5888 | 4445 | 7865 | 8375 | 9734 | 94875 |

**Supplementary material B: LBB equations**

The LBB method assumes that growth in size follows a von Bertalanffy growth function [1]:

$\boldsymbol{L}_{\boldsymbol{t}}\boldsymbol{=}\boldsymbol{L}_{\boldsymbol{\infty}}\boldsymbol{(1-}\boldsymbol{e}^{\boldsymbol{-K(t-}\boldsymbol{t}_{\boldsymbol{0}}\boldsymbol{)}}\boldsymbol{)}$ Eq. 1

*L_t_* is the fish size at time *t* (in year); *L_∞_* is asymptotic length; *K* is a constant of dimension year^-1^,expressing the rate at which *L_∞_* is approached; t is the age in year; and *t_0_* is a theoretical age corresponding to zero length.

Total mortality (*Z = M + F*) versus somatic growth rate (*K*) [2] is used to estimate the portion fully selected by the catch in number of lengths:

$N_{L}=N_{L_{start}}\left( \frac{L_{\infty}-L}{L_{\infty}-L_{start}} \right)^{Z/K}$ for *L ˃ L_start_* and *L ˂ L_∞_* Eq. 2

where *N_L_* is the number of survivors to the length *L*, *N_Lstart_* is the number at length *L_start_* with full selection, from which all specimens encountering the gear are retained. If there is no fishing, *Z/K* becomes *M/K, L_start_* is zero, and *N_Lstart_* can be set to 1 [3,4]. However, Eq. 3 will allow Eq. 2 to be completed in terms of the catch in numbers that is subject to partial selection is a function of the selectivity of the gear (lengths affected by partial selection):

$SL=\frac{1}{1+e^{-\propto\left( L-L_{c} \right)}}$ Eq.3

where *S_L_* is the fraction of individuals that are retained by the gear at length *L, L_c_* is the length at first capture, and α describes the steepness of the ogive [5]. Thus, the combination of Eq. 2 and 3 leads to the following equation [4]:

$N_{L_{i}}=N_{L_{i-1}}\left( \frac{L_{\infty}-L_{i}}{L_{\infty}-L_{i-1}} \right)^{\frac{M}{K}+\frac{F}{K}S_{L_{i}}}$ and $C_{L_{i}}= N_{L_{i}}S_{L_{i}}$ Eq. 4

where $N_{L_{i}}$ is the number of individuals in length class *L_i_*, $N_{L_{i-1}}$ is the number in the previous length class, $C_{L_{i}}$ represents the individuals that are vulnerable to the gear, and all other parameters are as described above [4]. Dividing both sides of Eq. 4 by their respective sums yields the version of the LBB equation that is actually fitted to the catch in numbers curve (Eq. 5) [5]:

$\frac{C_{L_{i}}}{\sum C_{L_{i}}}=\frac{N_{L_{i}}S_{L_{i}}}{\sum N_{L_{i}}S_{L_{i}}}$ Eq. 5

The length, *L_opt_* where unexploited cohort biomass is maximum is obtained, is given by Beverton [6] as:

$L_{opt}=L_{\infty}\frac{3}{3+\frac{M}{K}}$ Eq. 6

Starting fishing at *L_c_opt_* results in a mean length of *L_opt_* for the catch and the exploits part of the population [7]:

$L_{C\_opt}=L_{\infty}\frac{2+{3F}/M}{\left( 1+F/M \right)\left( 3+M/K \right)}$ Eq. 7

From the basic equations presented above, an index of yield per recruit expressed as a function of the length at first capture *L_c_* is given by Beverton and Holt [8] as:

${Y'}/R=\frac{F/M}{1+F/M} \left( 1-{L_{C}}/{L_{\infty}} \right)^{M/K}\left( 1-\frac{3\left( 1-{L_{C}}/{L_{\infty}} \right)}{1+\frac{1}{M/{K\left( 1+F/M \right)}}}+\frac{3\left( 1-{L_{C}}/{L_{\infty}} \right)}{1+\frac{2}{M/{K\left( 1+F/M \right)}}}-\frac{\left( 1-{L_{C}}/{L_{\infty}} \right)^{3}}{1+\frac{3}{M/{K\left( 1+F/M \right)}}} \right)$ Eq. 8

An index of catch per unit effort (*CPUE’ /R*), representing the relative stock status, is then calculated by following equation:

$\frac{CPUE'}{R}=\frac{{Y'}/R}{F/M}=\frac{1}{1+F/M}\left( 1-{L_{c}}/{L_{\infty}} \right)^{M/K}\left( 1-\frac{3\left( 1-{L_{c}}/{L_{\infty}} \right)}{1+\frac{1}{M/K+F/K}}+\frac{3\left( 1-{L_{c}}/{L_{\infty}} \right)^{2}}{1+\frac{2}{M/K+F/K}}-\frac{\left( 1-{L_{c}}/{L_{\infty}} \right)^{3}}{1+\frac{3}{M/K+F/K}} \right)$ Eq. 9

By setting *F* as 0, the relative biomass level of unexploited state could be obtained in Eq. 10:

$\frac{B_{0}'>L_{c}}{R}=\left( 1-{L_{c}}/{L_{\infty}} \right)^{M/K}\left( 1-\frac{3\left( 1-{L_{c}}/{L_{\infty}} \right)}{1+\frac{1}{M/K}}+\frac{3\left( 1-{L_{c}}/{L_{\infty}} \right)^{2}}{1+\frac{2}{M/K}}-\frac{\left( 1-{L_{c}}/{L_{\infty}} \right)^{3}}{1+\frac{3}{M/K}} \right)$ Eq.10

where *B’_0_ > L_c_* represents the exploitable fraction (*> L_c_*) of the unfished biomass (*B_0_*). According to Beverton and Holt [8], an index of relative biomass depletion for the exploited part of the population *B/B_0_* can be obtained by:

$\frac{B}{B_{0}}=\frac{{CPUE'}/R}{\frac{{B'}_{0}>L_{c}}{R}}$ Eq. 11

Using the *B/B_0_,*  *B/B_msy_* , *L_c_/L_m_* and *L_mean_/L_opt_* indicators, the status of a stock can be defined [5,9,10] as: "healthy" if *B/B_msy_* ≧ 1, "slightly overﬁshed" if 0.8 ≦ *B/B_msy_* < 1, "overﬁshed" if 0.5 ≦ *B/B_msy_* < 0.8, "grossly overﬁshed" if 0.2 ≦ *B/B_msy_* < 0.5, "collapsed" if *B/B_msy_* < 0.2 and if *B/B_0_* (0.4 - 0.5) as the reference limit of the biomass of a stock.

**Supplementary material C: Results of ANOVA tests**

Table 2: Results of ANOVA tests between mean annual length (TL; cm) of Five species of small pelagic fish exploited.

| Species | ANOVA test | | |
| --- | --- | --- | --- |
|  | *F-value* | *df* | *p-value* |
| *Scomber colias* | 1415 | 1 | <0.05 |
| *Mugil cephalus* | 7832 | 1 | <0.05 |
| *Sardina pilchardus* | 20.9 | 1 | <0.05 |
| *Ethmalosa fimbriata* | 8068 | 1 | <0.05 |
| *Trachurus trecae* | 107 | 1 | <0.05 |

**Supplementary material D: Results of LBB models**

1. **LBB results for *Scomber colias*, stock Scomber_senegal, 2004-2018**

**-----------------------------------------------------------------------**

**General reference points [median across years]:**

Linf prior= 48, SD=0.48 cm (user-defined), Lmax=49, median Lmax=49

Z/K prior = 1.1, SD=0.16, M/K prior=1.5, SD=0.15

Lc prior = 24.5, SD=2.4 cm, alpha prior=23.9, SD=2.4, Lm50=20 cm

Linf = 50.8 (50.2-51.4) cm

Lopt = 35 cm, Lopt/Linf=0.68

Lc_opt = 25 cm, Lc_opt/Linf=0.49, Lmean if F=M 35.5 cm

M/K = 1.4 (1.2-1.59)

F/M = 0.159 (0.0697-0.343), F/K=0.215 (0.0835-0.387), Z/K=1.62 (1.53-1.8)

B/B0 = 0.82 (0.15-1.9), B/B0 F=M Lc=Lc_opt 0.37

Y/R' = 0.024 (0.0051-0.054), Y/R' F=M Lc=Lc_opt 0.051

**-----------------------------------------------------------------------**

**Estimates for 2018 (mean of last 3 years with data):**

Lc50 = 30.6 (30.2-30.9) cm, Lc/Linf=0.6 (0.59-0.61)

Lc95 = 37, alpha=0.46 (0.447-0.473)

Lmean/Lopt= 1.1, Lc/Lc_opt=1.2, L95th=48 cm, L95th/Linf=0.94, Mature=96%

F/M = 0.75 (0.52-1), F/K=1.1 (0.86-1.3), Z/K=2.7 (2.5-2.9)

Y/R' = 0.02 (0.0091-0.033)

B/B0 = 0.64 (0.12-1.4), best LF fit year 2005=0.755 (0.16-1.6)

B/Bmsy = 1.7 (0.32-3.7), selected B/B0 2018 = 0.64 (0.12-1.4)


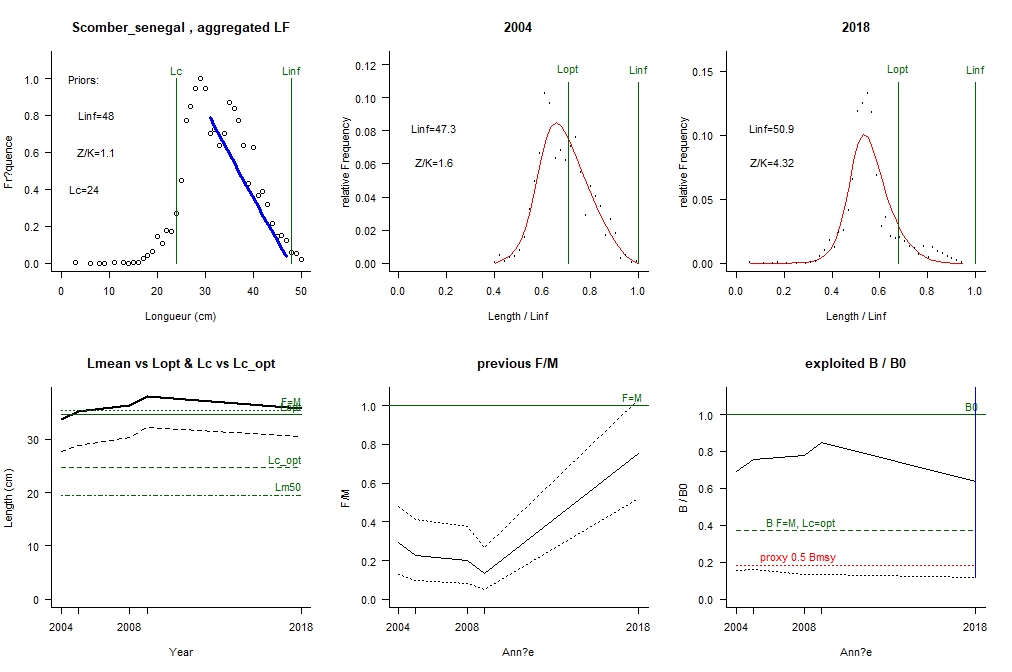


1. **LBB results for *Ethmalosa fimbriata*, stock Ethmalose_senegal, 2004-2019**

**-----------------------------------------------------------------------**

**General reference points [median across years]:**

Linf prior= 35, SD=0.35 cm (user-defined), Lmax=37, median Lmax=37

Z/K prior = 3, SD=0.34, M/K prior=1.5, SD=0.15

F/K prior = 1.46 (wide range with tau=4 in log-normal distribution)

Lc prior = 18.9, SD=1.9 cm, alpha prior=53.4, SD=5.3, Lm50=NA cm

Linf = 37.2 (36.7-37.8) cm

Lopt = 23 cm, Lopt/Linf=0.63

Lc_opt = 21 cm, Lc_opt/Linf=0.57, Lmean if F=M 23.4 cm

M/K = 1.78 (1.53-2.1)

F/M = 2.83 (2.39-3.72), F/K=5.46 (5.01-6.01), Z/K=7.38 (7.02-7.85)

B/B0 = 0.14 (0.1-0.19), B/B0 F=M Lc=Lc_opt 0.36

Y/R' = 0.019 (0.013-0.026)(reduced: B/B0<0.25),Y/R' F=M Lc=Lc_opt 0.035

**-----------------------------------------------------------------------**

**Estimates for 2019 (mean of last 3 years with data):**

Lc50 = 19.1 (19-19.2) cm, Lc/Linf=0.52 (0.52-0.52)

Lc95 = 21.5, alpha=1.25 (1.22-1.29)

Lmean/Lopt= 0.92, Lc/Lc_opt=0.9, L95th=35 cm, L95th/Linf=0.95, Mature=NA%

F/M = 5.8 (4.7-7), F/K=10 (9.5-11), Z/K=12 (11-13)

Y/R' = 0.013 (0.0095-0.017)(reduced because B/B0 < 0.25)

B/B0 = 0.084 (0.064-0.11), best LF fit year 2015=0.133 (0.089-0.19)

B/Bmsy = 0.24 (0.18-0.31), selected B/B0 2019 = 0.084 (0.064-0.11)


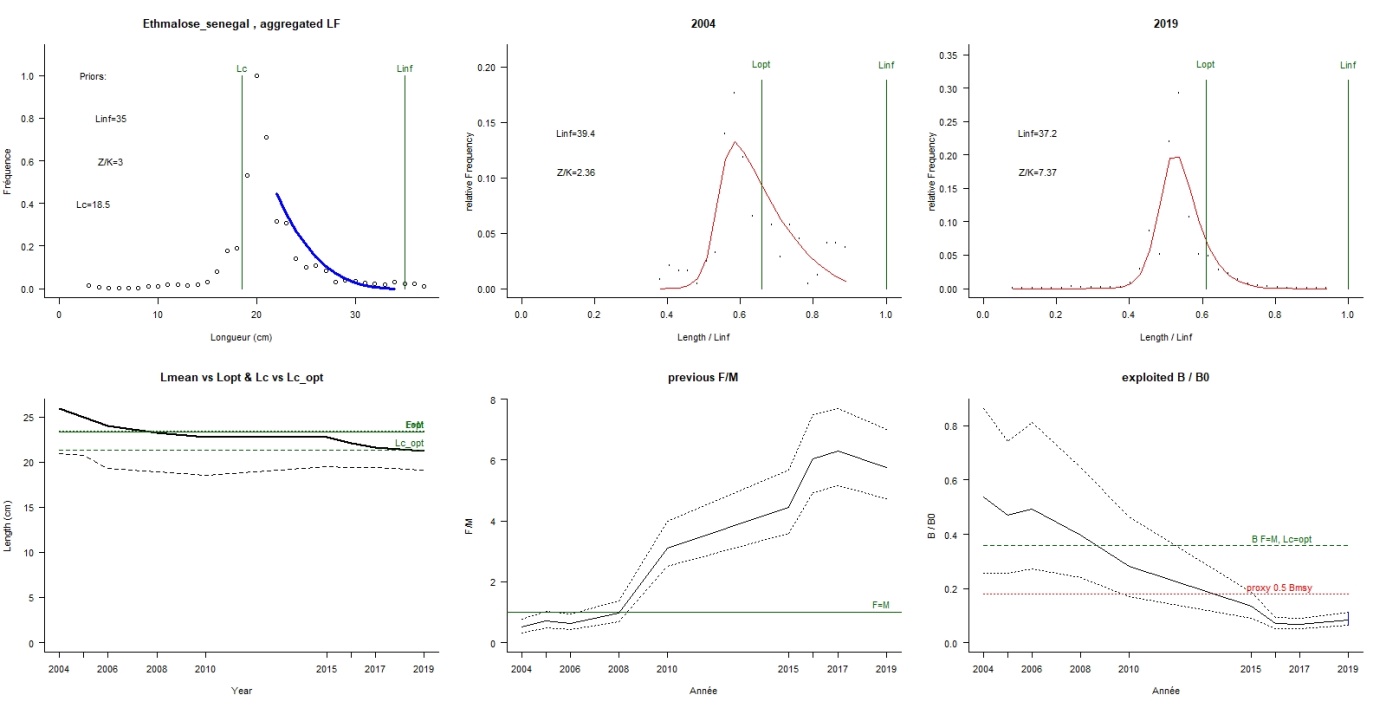


1. **LBB results for *Mugil cephalus*, stock Mugil_senegal, 2005-2019**

**-----------------------------------------------------------------------**

**General reference points [median across years]:**

Linf prior= 66, SD=0.66 cm (user-defined), Lmax=70, median Lmax=70

Z/K prior = 3.4, SD=0.23, M/K prior=1.5, SD=0.15

Lc prior = 21.9, SD=2.2 cm, alpha prior=87.9, SD=8.8, Lm50=27.5 cm

Linf = 69.8 (69-70.9) cm

Lopt = 47 cm, Lopt/Linf=0.67

Lc_opt = 43 cm, Lc_opt/Linf=0.61, Lmean if F=M 33.4 cm

M/K = 1.5 (1.23-1.74)

F/M = 3.01 (2.41-4.03), F/K=4.65 (4.24-5.11), Z/K=6.19 (5.94-6.48)

B/B0 = 0.076 (0.053-0.11), B/B0 F=M Lc=Lc_opt 0.37

Y/R' = 0.0096 (0.0067-0.013)(reduced: B/B0<0.25),Y/R' F=M Lc=Lc_opt 0.046

**-----------------------------------------------------------------------**

**Estimates for 2019 (mean of last 3 years with data):**

Lc50 = 21.2 (21.2-21.3) cm, Lc/Linf=0.3 (0.3-0.3)

Lc95 = 23.6, alpha=1.23 (1.19-1.28)

Lmean/Lopt= 0.61, Lc/Lc_opt=0.5, L95th=65.7 cm, L95th/Linf=0.94, Mature=32%

F/M = 4.3 (3.5-5.4), F/K=6.6 (6.1-7), Z/K=8.1 (7.8-8.4)

Y/R' = 0.0053 (0.0039-0.007)(reduced because B/B0 < 0.25)

B/B0 = 0.047 (0.035-0.062), best LF fit year 2011=0.365 (0.15-0.66)

B/Bmsy = 0.13 (0.094-0.17), selected B/B0 2019 = 0.047 (0.035-0.062)


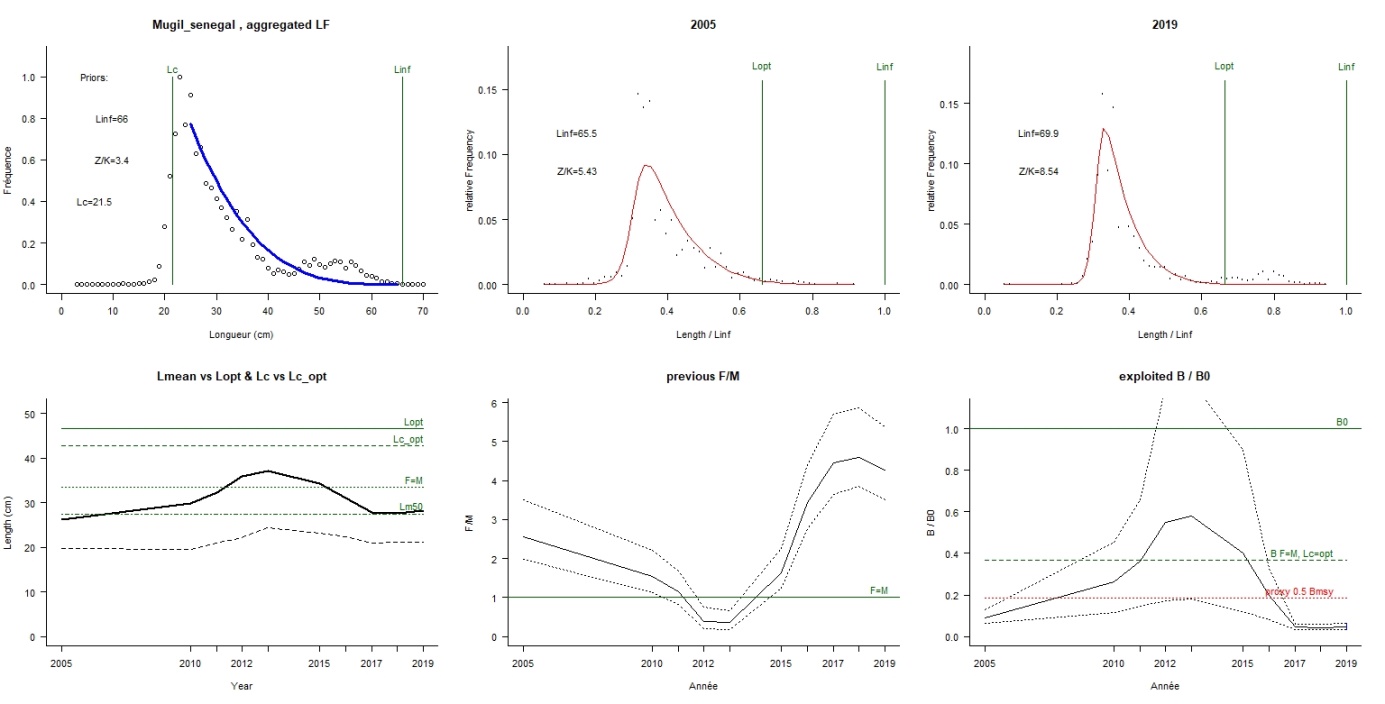


1. **LBB results for *Sardina pilchardus*, stock Sardina_senegal, 2005-2019**

**-----------------------------------------------------------------------**

**General reference points [median across years]:**

Linf prior= 30, SD=0.3 cm (user-defined), Lmax=31, median Lmax=30.5

Z/K prior = 2.6, SD=0.21, M/K prior=1.5, SD=0.15

F/K prior = 1.15 (wide range with tau=4 in log-normal distribution)

Lc prior = 22.4, SD=2.2 cm, alpha prior=32.1, SD=3.2, Lm50=15.4 cm

Linf = 30.5 (30.2-31) cm

Lopt = 20 cm, Lopt/Linf=0.67

Lc_opt = 18 cm, Lc_opt/Linf=0.59, Lmean if F=M 24.4 cm

M/K = 1.49 (1.22-1.75)

F/M = 1.89 (1.22-2.59), F/K=2.99 (2.6-3.47), Z/K=4.37 (4.09-4.73)

B/B0 = 0.28 (0.17-0.4), B/B0 F=M Lc=Lc_opt 0.39

Y/R' = 0.042 (0.022-0.062), Y/R' F=M Lc=Lc_opt 0.044

**-----------------------------------------------------------------------**

**Estimates for 2019 (mean of last 3 years with data):**

Lc50 = 18.5 (18.3-18.6) cm, Lc/Linf=0.61 (0.61-0.62)

Lc95 = 20.7, alpha=1.32 (1.27-1.36)

Lmean/Lopt= 0.82, Lc/Lc_opt=1, L95th=23.7 cm, L95th/Linf=0.78, Mature=100%

F/M = 13 (-30-43), F/K=10 (9-12), Z/K=11 (9.9-12)

Y/R' = 0.032 (-0.084-0.12)

B/B0 = 0.04 (-0.055-0.12), best LF fit year 2015=0.25 (0.1-0.46)

B/Bmsy = 0.1 (-0.14-0.3), selected B/B0 2019 = 0.04 (-0.055-0.12)


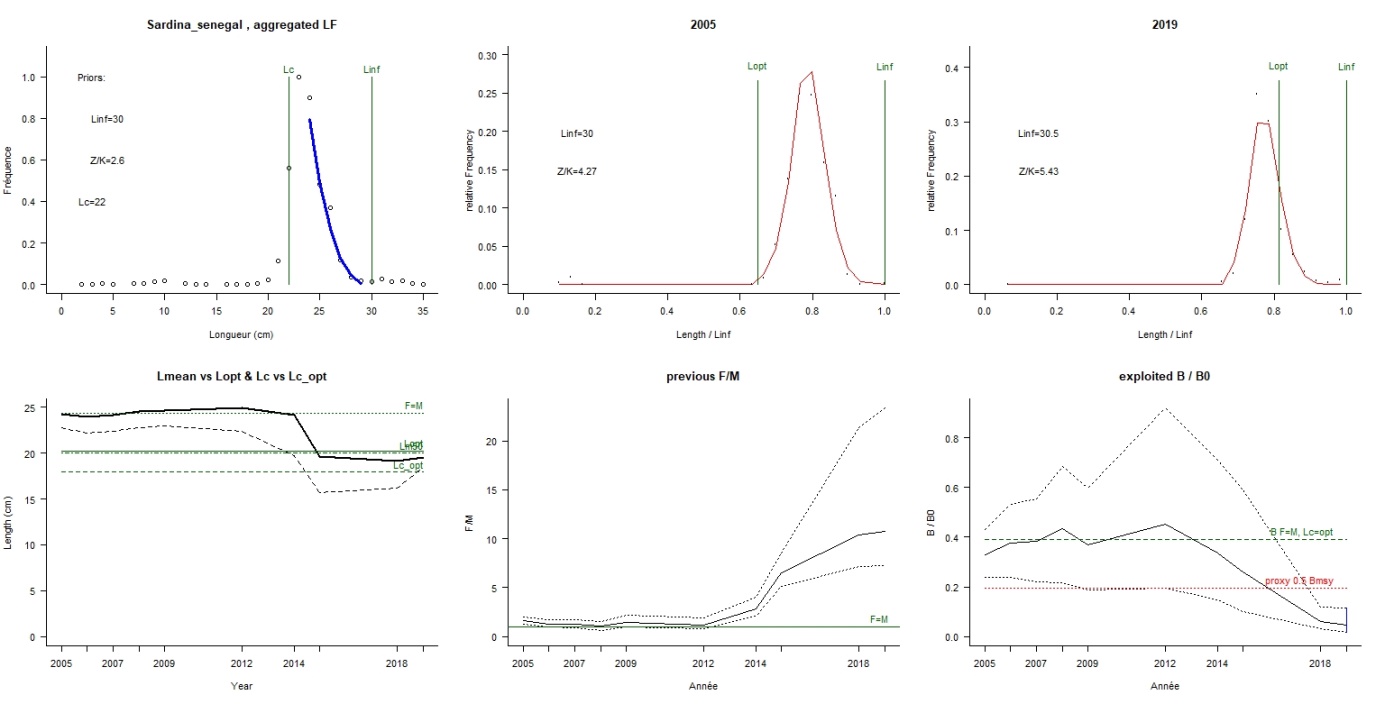


1. **LBB results for *Trachurus trecae*, stock Trachurus_senegal, 2004-2019**

**-----------------------------------------------------------------------**

**General reference points [median across years]:**

Linf prior= 35, SD=0.35 cm (user-defined), Lmax=39, median Lmax=39

Z/K prior = 1, SD=0.68, M/K prior=1.5, SD=0.075(user-defined)

F/K prior = 0.3 (wide range with tau=4 in log-normal distribution)

Lc prior = 22.9, SD=2.3 cm, alpha prior=31.1, SD=3.1, Lm50=15 cm

Linf = 37.1 (36.6-37.5) cm

Lopt = 25 cm, Lopt/Linf=0.67

Lc_opt = 21 cm, Lc_opt/Linf=0.56, Lmean if F=M 26.9 cm

M/K = 1.46 (1.27-1.57)

F/M = 0.905 (0.711-1.16), F/K=1.55 (1.23-1.88), Z/K=3.24 (2.95-3.5)

B/B0 = 0.42 (0.25-0.57), B/B0 F=M Lc=Lc_opt 0.37

Y/R' = 0.023 (0.019-0.027), Y/R' F=M Lc=Lc_opt 0.048

**-----------------------------------------------------------------------**

**Estimates for 2019 (mean of last 3 years with data):**

Lc50 = 24.4 (24.2-24.6) cm, Lc/Linf=0.65 (0.65-0.65)

Lc95 = 28.1, alpha=0.789 (0.763-0.817)

Lmean/Lopt= 1.1, Lc/Lc_opt=1.2, L95th=35 cm, L95th/Linf=0.93, Mature=99%

F/M = 1 (0.86-1.3), F/K=1.5 (1.3-1.8), Z/K=3 (2.8-3.2)

Y/R' = 0.032 (0.023-0.044)

B/B0 = 0.52 (0.28-0.91), best LF fit year 2019=0.525 (0.28-0.91)

B/Bmsy = 1.4 (0.77-2.5), selected B/B0 2019 = 0.52 (0.28-0.91)


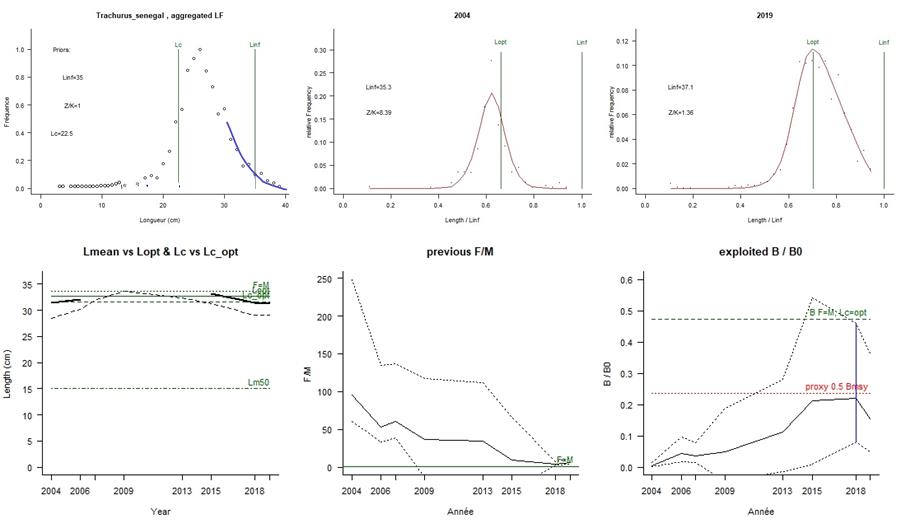

Supplement: S1 File — (DOCX) [file pone.0279768.s001.docx]
